# Ex Vivo Characterization and In Vivo Nasal Delivery of Ropinirole-Loaded PEO-b-PCL/Tween 80/β-Cyclodextrin Systems in C57BL/6J Mice

**DOI:** 10.3390/molecules31091405

**Published:** 2026-04-23

**Authors:** Elmina-Marina Saitani, Paraskevi Papakyriakopoulou, Evangelos Balafas, Dimitrios E. Damalas, Nikolaos Kostomitsopoulos, Stergios Pispas, Natassa Pippa, Nikolaos Thomaidis, Georgia Valsami

**Affiliations:** 1 Section of Pharmaceutical Technology, Department of Pharmacy, School of Health Sciences, National and Kapodistrian University of Athens, Panepistimiopolis Zografou, 15771 Athens, Greece; e.saitani@pharm.uoa.gr (E.-M.S.); ppapakyr@pharm.uoa.gr (P.P.); natpippa@pharm.uoa.gr (N.P.); 2 Laboratory Animal Facility, Centre of Clinical and Experimental Surgery and Translational Research, Biomedical Research Foundation, Academy of Athens, 11527 Athens, Greece; vbalafas@bioacademy.gr (E.B.); nkostom@bioacademy.gr (N.K.); 3 Laboratory of Analytical Chemistry, Department of Chemistry, National and Kapodistrian University of Athens, Panepistimioupolis Zografou, 15771 Athens, Greece; dimdamalas@chem.uoa.gr (D.E.D.); ntho@chem.uoa.gr (N.T.); 4 Theoretical and Physical Chemistry Institute, National Hellenic Research Foundation, 48 Vassileos Constantinou Avenue, 11635 Athens, Greece; pispas@eie.gr

**Keywords:** ropinirole hydrochloride, intranasal drug delivery, colloidal dispersion, ex vivo permeation studies, pharmacokinetics, CNS targeting

## Abstract

Intranasal administration is a promising drug delivery route enabling precise and rapid central nervous system targeting. In our previous work, twelve hybrid colloidal dispersions were developed, consisting of synthetic poly(ethylene-oxide)-b-poly(ε-caprolactone) (PEO-b-PCL) block copolymers with an increasing proportion of the hydrophobic PCL segment, Tween 80 (Tw80) and β-cyclodextrin derivatives (βCD), either methyl-β-CD (MβCD) or hydroxy-propyl-β-CD (HPβCD) for IN delivery of ropinirole hydrochloride (RH). Colloidal dispersions were prepared at different weight ratios (system/RH equal to 10:1 and 10:5), characterized and evaluated in vitro. The aim of this study is to evaluate the ex vivo permeation through rabbit nasal mucosa and determine the pharmacokinetic parameters of RH, when administered intranasally as a colloidal dispersion, compared with oral and intranasal RH solutions in C57BL/6J mice. Ex vivo permeation studies showed that all formulations significantly enhanced RH permeation compared to the pure RH solution (0.5 mg/mL, pH 5.6). Among them, F4 [(PEO-b-PCL_1_/Tw80/HPβCD)/RH 10:5] was selected for further investigation. Pharmacokinetic analysis showed that F4 significantly enhanced both systemic and brain exposure of RH, achieving higher serum AUC and C_max_ values, despite a 3-fold lower administered dose compared to the oral dose. It showed high systemic (F_rel(Serum)_ = 1815%) and brain (F_rel(Brain)_ = 363%) relative bioavailability compared with oral administration, underscoring its potential as an intranasal delivery system for efficient CNS targeting.

## 1. Introduction

Parkinson’s disease (PAD) is a progressive neurodegenerative disorder characterized by the selective degeneration of dopaminergic neurons in the substantia nigra pars compacta, leading to dopamine depletion in the striatum [1,2]. PAD is manifested by both motor symptoms—including bradykinesia, resting tremor, muscular rigidity, and postural instability—and non-motor symptoms such as cognitive impairment, depression, anxiety, sleep disturbances, autonomic dysfunction, hyposmia, and pain [3].

Current therapies for PAD typically combine pharmacological treatments with non-pharmacological interventions to manage the symptoms, especially the motor ones. However, the therapeutic regimen for PAD fails to reverse the disease progression [4]. These therapeutic limitations have steered research toward innovative delivery strategies and alternative routes of administration to enhance central nervous system (CNS) targeting.

Intranasal (IN) administration has emerged as a promising drug delivery route, offering direct nose-to-brain transport, bypassing the blood–brain barrier (BBB) and first-pass metabolism, reducing enzymatic degradation, minimizing systemic exposure and peripheral side effects, and enabling more precise, rapid, and patient-friendly CNS targeting [4,5].

Recent studies have demonstrated the potential of IN systems for PAD management [6,7,8,9,10]. Lin et al. [8] designed a thermoresponsive gel containing poloxamer 407, poloxamer 188, polyethylene glycol 6000, and hydroxypropyl-β-cyclodextrin (HPβCD) for the IN delivery of the antiparkinsonian agent rhynchophylline. Imran et al. [6] formulated a mucoadhesive microemulsion for silymarin, which improved behavioral and biochemical outcomes while reducing inflammatory markers in treated rats. Kim [7] formulated a novel IN strategy aimed at enhancing mitochondrial function and protecting against PAD-related neurodegeneration. Sridhar et al. [10] developed a mucoadhesive thermosensitive gel by combining P407 with chitosan to increase the bioavailability and brain uptake of selegiline hydrochloride, an established anti-Parkinson’s medication.

In this context, in our previous research, twelve hybrid colloidal dispersions were designed and developed, consisting of synthetic, biocompatible block copolymers poly(ethylene-oxide)-b-poly(ε-caprolactone) (PEO-b-PCL) with an increasing proportion of the hydrophobic PCL block, Tween 80 (Tw80) and β-cyclodextrin derivatives (βCD), either methyl-β-CD (MβCD) or hydroxy-propyl-β-CD (HPβCD) for the possible IN delivery of ropinirole hydrochloride (RH). PEO-b-PCL_1_, PEO-b-PCL_2_, and PEO-b-PCL_3_ block copolymers were produced, containing 15%, 30%, and 53% wt of the PCL hydrophobic component, respectively. More specifically, PEO-b-PCL/Tw80/MβCD/RH and PEO-b-PCL/Tw80/HPβCD/RH colloidal dispersions were prepared at different weight ratios (system/RH equal to 10:1 and 10:5) (formulations F1–F12) [11]. Table 1 presents all the formulations prepared using the synthetic PEO-b-PCL block copolymers.

Previous studies revealed that systems containing PEO-b-PCL_1_ and PEO-b-PCL_2_ polymers presented colloidal stability that can be attributed to the steric repulsion of the hydrophilic polymeric blocks, which is a property provided by the hydrophilic corona of the PEO chains of each polymer. Based on the MTT assay studies, all samples showed dose-dependent toxicity on the HEK293 cell lines, with the systems containing HPβCD considered non-toxic, even at high concentrations of 100, 200, and 300 μg/mL. In vitro diffusion experiments under pH and temperature conditions that mimic those of the human nasal cavity consistently demonstrated an effective release of RH from the formulations, with a mass balance exceeding 90% in most cases. Particularly, systems containing a 5 mg/mL concentration of RH and HPβCD exhibited superior in vitro release characteristics resembling those of the RH solution (0.5 mg/mL, pH 5.6).

RH is classified as a BCS class III drug (high water solubility and low permeability) [12], which resulted in no statistically significant differences between the release profiles of the prepared formulations. Further investigation through ex vivo permeation experiments was performed to corroborate these findings. Based on our findings, the optimal formulation F4 [(PEO-b-PCL_1_/Tw80/HPβCD)/RH 10:5] was selected for further comparative in vivo evaluation in black C57BL/6J mice.

In this context, the aim of this study is to develop and evaluate a biocompatible hybrid colloidal system composed of PEO-b-PCL, HPβCD, and Tw80 for the intranasal (IN) delivery of RH in mice. To the best of our knowledge, this is the first study to investigate both the ex vivo permeation across rabbit nasal mucosa and the in vivo pharmacokinetic (PK) profile of RH delivered via a PEO-b-PCL/CD/surfactant ternary system, in comparison with RH solution administered intranasally or orally. This approach addresses the current lack of data on such ternary systems for intranasal RH delivery and is expected to enable dose reduction while enhancing systemic exposure and brain bioavailability, potentially reducing adverse effects and offering an alternative non-invasive route of administration.

Thus, the PK parameters of RH when administered intranasally as a colloidal dispersion (F4) for possible nose-to-brain delivery, in comparison to the *per os* (PO) and IN administration of control RH solution in C57BL/6J mice, were determined. Brain and serum distribution PKs of the drug were studied applying by sparse-sampling non-compartmental analysis (NCA) using Phoenix^®^ 8.3.5 software (Certara, Randor, PA, USA). The IN bioavailability relative to PO administration (F_rel_) was calculated by comparing the respective Areas Under the Curve (AUCs) after IN and PO administration [13].

## 2. Results and Discussion

### 2.1. RH’s Ex Vivo Mucosal Permeation from the Hybrid Formulations

**Figure 1:**
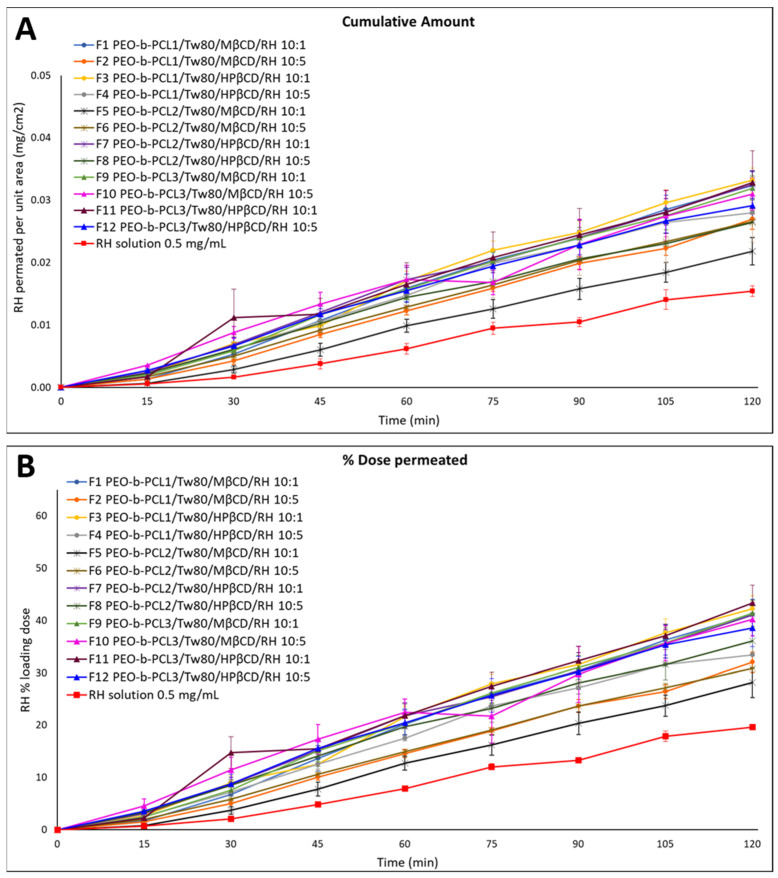
Permeation profiles for colloidal dispersions of RH PEO-b-PCL ternary systems (F1–F12) through rabbit nasal mucosa for formulations at weight ratios of 10:1 and 10:5 (loading doses equal to 0.05 mg of RH) in comparison to RH solution (0.5 mg/mL, pH 5.6). Results are expressed as (**A**) quantity permeated per unit area (mean ± SE, n = 4) and (**B**) % loading dose permeated for the tested formulation (mean ± SE, n = 4).

Figure 1 and Table 2 present the results of the ex vivo permeation experiments conducted with the RH loading dose equal to 0.05 mg. The flux across the nasal mucosa barrier (J_NM_) for all the tested formulations varied from 1.9 × 10^−4^ ± 0.8 × 10^−5^ to 2.9 × 10^−4^ ± 0.9 × 10^−5^ μg/cm^2^/min, while the apparent permeability (*P_app_*) ranged from 0.38 to 0.58 cm/min. The nasal mucosa barrier retained between 3.35 ± 0.34% and 17.12 ± 1.14% of the initial loading dose of RH. Table S1 (Supplementary Material) details the percentage of mass balance of RH in each formulation, the percentage of the loading dose retained by the nasal mucosa barrier, the J_NM_, the *P_app_* and the R-square values from the regression analysis of permeated amount per unit area over time. The consistently high mass balance values across all formulations and RH solution confirmed the chemical stability of RH under the experimental conditions applied.

#### 2.1.1. Effect of System/RH Ratio and CD Type on RH Mucosal Permeation

For the RH solution, the percentage of the loading dose that permeated through the nasal mucosa was 19.62 ± 0.53%. All tested formulations (F1–F12 colloidal dispersions) displayed significantly higher RH permeation compared to the RH solution (*p* < 0.05, 95% CI). More precisely, the % amount of RH that permeated across the rabbit nasal mucosa was 44% to 115% higher than the corresponding percentage that permeated from the pure RH solution at the end of the experiment. These differences were even more substantial at earlier time points, with increases reaching up to 539% and 260% at 15 and 45 min, respectively. The enhanced permeation observed with these formulations is likely attributed to the presence of CDs, which are known to interact with the nasal epithelium [14,15]. Such interactions may disrupt tight junctions, thereby enhancing RH’s permeation. Additionally, CDs can interact with membrane components such as phospholipids and cholesterol, increasing membrane fluidity and further enhancing the passage of RH and other active pharmaceutical ingredients (APIs) [15].

In line with the in vitro release results, most formulations containing HPβCD demonstrated higher % RH permeation across the nasal mucosa compared to those with MβCD. The most significant differences (*p* < 0.05, 95% CI) were observed between F5 (containing PEO-b-PCL_2_, MβCD, and RH at 1 mg/mL) and F7 (containing PEO-b-PCL_2_, HPβCD, and RH at 1 mg/mL), with RH permeation from F7 reaching up to 275% higher than F5 (Figure 1, Table 2). The overall superior performance of most HPβCD-based formulations aligns also with findings from our previous study [16,17].

Although MβCD is widely known to exert stronger effects on the epithelial barrier by transiently opening the tight junctions of the nasal epithelium [18], HPβCD is considered less disruptive for mucosa. In this study, despite RH’s high aqueous solubility—which limits the relevance of CD-mediated solubilization—the HPβCD-based systems showed superior permeation. This suggests that enhancement is not driven by increased solubility but rather by microstructural and interfacial phenomena. HPβCD likely formed more homogeneous colloidal assemblies with the polymer (PEO-b-PCL) and surfactant (Tw80), favoring a stable dispersion, improved wetting at the mucosal interface, and a dynamic complexation–decomplexation equilibrium that sustained drug flux. This hypothesis is also supported by DSC data outlined in our previous study [11], which revealed variations in thermal behavior depending on the type of CD used. Specifically, the nature of the substitutions groups and the differing hydrophobicity of the two βCD derivatives influence the balance of hydrophilic and hydrophobic interactions within the hybrid system (polymer, Tw80 and CD). The DSC results indicated the potential formation of an inclusion complex. Specifically, the hydrophobic PEO chains of the polymer and the hydrophobic subunits of Tw80 interact with the lipophilic core of CD via van der Waals forces. Moreover, the hydrophilic surface of CD can form hydrogen bonds with the PEO chains of the PEO-b-PCL block copolymer and the ethylene oxide subunits of the surfactant.

#### 2.1.2. Effect of Polymer Type on RH Mucosal Permeation

Figures S1–S3 (Supplementary Material) demonstrate the permeation profiles of RH-loaded colloidal dispersions containing PEO-b-PCL_1_, PEO-b-PCL_2_ and PEO-b-PCL_3_, respectively. A comparison of RH permeation profiles among formulations containing different types of PEO-b-PCL block copolymers revealed no statistically significant differences (*p* > 0.05, 95% CI) within the formulations containing HPβCD (F3 vs. F7 vs. F11 and F4 vs. F8 vs. F12) during the experiment. In contrast, significant differences (*p* < 0.05, 95% CI) were observed in specific comparisons, notably between F1 and F5 at the end of the experiment (F1 > F5), and between F10 and F6 at 60 and 120 min (F10 > F6) (*p* < 0.05, 95% CI).

Several studies have explored the potential nose-to-brain delivery of RH using hybrid nanoparticles [19,20,21,22]. The main advantage of these systems lies in their multi-component composition, which combines materials of diverse origins—such as polymers, CDs, and lipids. This combination improves RH permeability, allowing for both site-specific targeting and controlled drug release. Overall, these findings indicate a significant increase in RH permeation across rabbit nasal mucosa from all the tested formulations compared to the pure RH solution. This improved permeation is likely attributed to the presence of excipients (PEO-b-PCL block copolymers, Tw80, and CDs), which may interact with the nasal epithelial barrier to modulate its integrity and facilitate drug transport. These findings suggest a synergistic effect arising from the combined action of these components. Among all tested formulations, those containing HPβCD were more effective than those with MβCD in enhancing RH permeation, emerging as the most promising ones for further investigation. Further studies were implemented to evaluate the in vivo serum and brain PK profiles of the most promising formulations after nasal administration in mice.

### 2.2. Nasal Administration in C57BL/6J Mice

The F4 nasal formulation used in this study has already been thoroughly characterized in terms of its physicochemical and morphological properties in our previous work [11]. In brief, the (PEO-b-PCL_1_/Tw80)/HPβCD system demonstrated a mean hydrodynamic diameter of approximately 99 nm, with a polydispersity index of 0.3. The measured zeta potential was close to neutral, suggesting that colloidal stability is predominantly governed by steric stabilization mechanisms. This stabilization is attributed to the presence of the hydrophilic PEO corona, which provides effective repulsive interactions between nanoparticles. Furthermore, thermal analysis revealed significant intermolecular interactions among the formulation constituents, which were further confirmed by high-resolution ultrasound spectroscopy measurements, indicating a high degree of structural integration within the hybrid system. Collectively, the physicochemical and morphological characterization showed that the developed hybrid nanocarrier exhibits well-defined structural organization and favorable physicochemical properties, arising from the synergistic integration of all the system’s components (polymer, surfactant and CD).

The F4 colloidal dispersion demonstrated favorable attributes for RH IN delivery, particularly in in vitro release and ex vivo permeation studies. Notably, F4 exhibited first-order release kinetics, with 92.37 ± 4.46% of the RH-loaded dose released within 4 h. The ex vivo permeation across the rabbit nasal mucosa barrier demonstrated a linear profile (R-square = 0.9950 ± 0.0006), leading to 33.4 ± 0.18% of the loaded dose being transported within 2 h [11].

During the in vivo experiments, both RH solutions (administered intranasally and orally) and the F4 formulation were delivered without any observed signs of distress or nasal bleeding. The administration process, conducted under inhalation anesthesia, lasted less than one minute. Following administration, the mice recovered promptly and exhibited normal behaviors such as free movement, social interaction, self-grooming, chasing, standing up, wagging the tail, and regular eating and drinking until sacrifice at pre-determined time points.

Furthermore, the FDA has established a no observed adverse effect level (NOAEL) for RH at 15 mg/kg/day in mice, based on the absence of uterine endometrial polyps in a 2-year study. In our work, the IN F4 colloidal dispersion was administered at 4 mg/kg, which is 3.8-fold lower than the mouse NOAEL. This confirms that the selected dose was well within recognized nonclinical safety margins, supporting its suitability and ethical justification of the chosen dose regimen.

### 2.3. RH UHPLC-TIMS-QTOF-MS Assay

The concentration of RH in biological samples was determined using the UHPLC TIMS-QTOF system. A high-confidence identification strategy was implemented based on a 4D analytical template established from reference standards, as illustrated in Figure S4 (Supplementary Material). This template integrated characteristic experimental evidence, including the accurate mass precursor ([M+H]^+^ at *m*/*z* 261.1605), the retention time (R_t_ = 4.2 min), a distinct ion mobility value (1/K_0_ = 0.775 V·s/cm^2^), and the MS/MS fragmentation pattern showing three characteristic qualifier ions at *m*/*z* 160.0758, 132.0807, and 114.1277 (Supplementary Material, Figure S4d). For a successful identification of RH in biological samples, strict criteria were applied. In addition to high mass accuracy (<5 mDa) and a precise isotopic profile (mSigma < 200), the detection of >2 qualifier ions (Supplementary Material, Figure S4c) and a consistent mobilogram (EIM) profile matching the reference standard were mandatory. The robustness of this identification confirms the presence of RH with enhanced selectivity across the complex matrices of mouse serum and brain tissue. Figure S4 (Supplementary Material) exemplifies the identification of RH in a brain sample, highlighting the different identification criteria showing excellent agreement with the values established from the reference standard analysis.

A linear relationship was revealed between the peak area ratios (RH peak area/ISTD peak area) and RH nominal concentration over the examined range (0.010–0.300 μg/mL). An overall correlation coefficient (R-square) of 0.9997 [±0.0127%, RSD (%)] and 0.9993 [±0.0399%, RSD (%)] was obtained from the serum and brain sets of calibration curves, respectively. The limit of detection (LOD) and lower limit of quantification (LLOQ) of RH in mouse serum were 0.0031 ± 0.0004 and 0.0092 ± 0.0124 μg/mL, respectively, while in the case of brain tissue they were 0.0031 ± 0.0002 and 0.0094 ± 0.0006 μg/mL, respectively. In all cases, back-calculated concentrations of the calibration standards were within ±15% of the nominal value and ±20% for the LLOQ.

### 2.4. Pharmacokinetic Profiles of RH After Intranasal and per os Administration

The combination of block copolymers with CDs has led to the development of a novel category of drug delivery systems characterized by unique morphological and functional characteristics [23], including excellent biocompatibility [24,25], enhanced drug-loading capacity [26,27], the ability to provide sustained or controlled drug release [24,28], improved colloidal stability [29], and superior permeability across biological barriers with targeted delivery to specific tissues or cells [25]. Therefore, it was worthwhile to investigate the systems developed from this combination to assess their effect on the PK behavior of RH, in the context of preclinical animal studies. The superiority of nasal delivery of RH has already been reported [9,30]. However, this is the first report to investigate the IN delivery of RH using a colloidal dispersion that combines synthetic and biocompatible block copolymers with CDs and surfactants.

**Figure 2:**
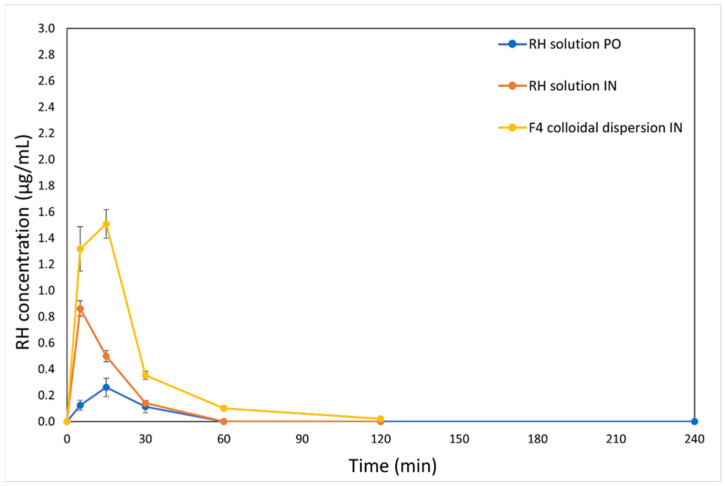
Serum concentration–time profiles of RH following intranasal administration, as RH solution (•), F4 colloidal dispersion (•), and oral administration as RH solution via gavage method (•). Data are expressed as mean ± SE, n = 5 for PO group, n = 5 for IN group of RH solution group, and n = 3–5 for the IN F4 group.

**Figure 3:**
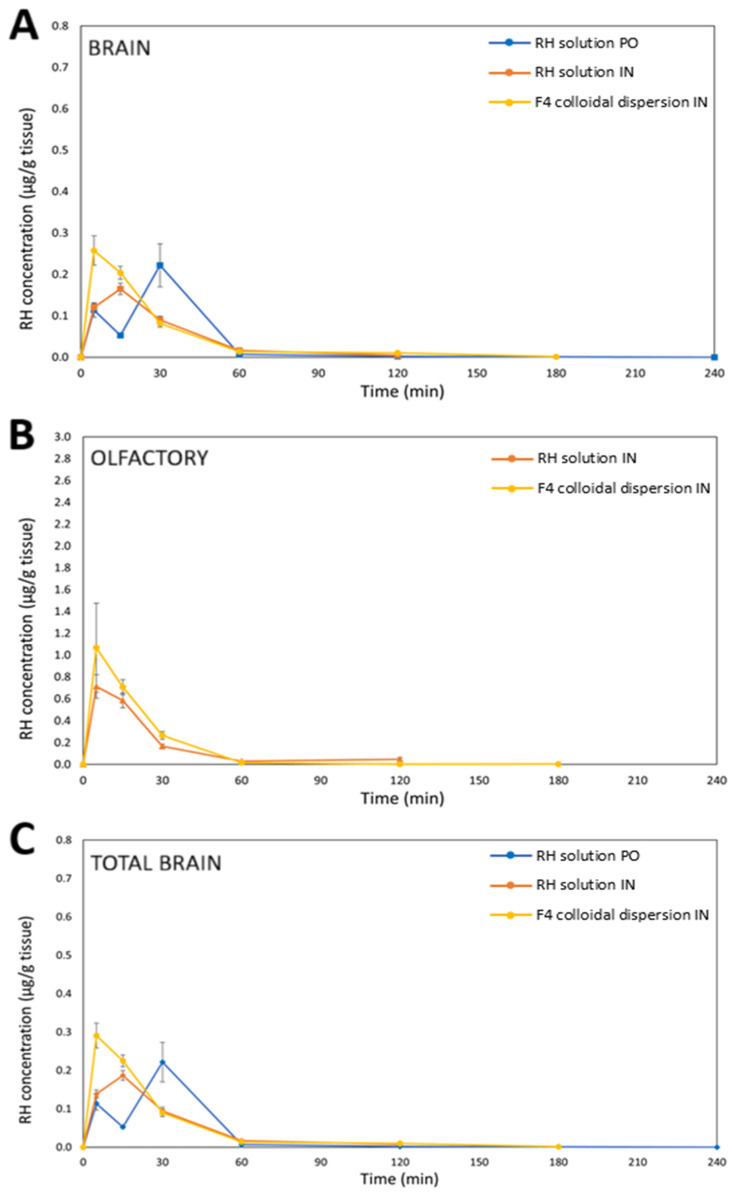
(**A**) Brain concentration–time profiles of RH following intranasal administration, as RH solution (▪), F4 colloidal dispersion (▪), and oral administration as RH solution via gavage method (▪); (**B**) olfactory concentration–time profiles of RH following intranasal administration, as RH solution (▲) and F4 colloidal dispersion (▲); and (**C**) Total brain concentration–time profiles of RH following intranasal administration, as RH solution (♦), F4 colloidal dispersion (♦) and oral administration as RH solution via gavage method (♦). Data are expressed as mean ± SE, n = 5 for PO group, n = 5 for IN group of RH solution, and n = 3–5 for IN F4 group.

Figure 2 and Figure 3 present the time-dependent concentration profiles of RH in both serum and brain tissue after IN and PO administration. It is noted that for the IN route, RH concentrations were determined separately in the olfactory bulbs and in the remaining brain tissue, whereas for the PO route, the brain levels were assessed as a whole. More specifically, Figure 3A–C display RH concentration over time in the brain, olfactory bulbs, and total brain, respectively.

The C_max_ values in serum differed significantly between the two routes of administration. Notably, RH delivered via the F4 formulation resulted in the highest C_max_, followed by the RH solution given intranasally, with the PO RH solution resulting in the lowest C_max_. However, the brain PK profiles (Figure 3) revealed only slight variations in C_max_ values between PO and IN delivery. It is important to note that IN administration was implemented using a dose three times lower than that of the PO route, yet it exhibited a superior PK profile—particularly in serum—underscoring the superiority of this route of administration.

Despite a simple plasma profile (C_max_ at 15 min), the brain displayed two distinct concentration maxima (at 5 min and 30 min) following oral administration of RH. As brains were perfused prior to sampling, the early rise cannot be attributed to residual blood. Instead, this pattern may reflect an initial rapid entry which is followed by a slower secondary uptake, leading to a subsequent increase in total brain concentrations even as plasma levels decline. To our knowledge, such a brain distribution pattern of RH has not been previously reported. However, given the limited variability of samples at 15 min and 30 min, the possibility of this double peak cannot be excluded and warrants further investigation. Notably, previous studies reported longer T_max_ values following oral administration. For example, Ali and Al-Akkam [31] observed a T_max_ of approximately 90 min in serum after the oral administration of an RH solution in rats, while Mantry and Balaji [32] reported a T_max_ of 112 min in rabbits receiving a 5 mg RH tablet. Dudhipala and Gorre [33] reported significantly different T_max_ values of RH following oral administration in rats, attributed to variations in the excipients and formulations used. Specifically, the T_max_ was 4 h after *per os* administration of RH solid lipid nanoparticles and RH nanostructured lipid carriers, and 6 h after the administration of an RH suspension formulated with Tween 20 (1% *w*/*v*) as the suspending agent. These variations compared to the present findings are likely due to differences in animal models, formulations, and experimental protocols.

The IN administration of both F4 and RH solutions showed quick systemic absorption, indicating effective transport through the nasal mucosa, which is beneficial for fast-acting therapeutic use. Both the RH solution and F4 formulation, when administered intranasally, achieved quick absorption, with C_max_ values reaching within 5 min (RH solution) and 15 min (F4) in serum. However, for F4, RH concentrations in serum at 5 and 15 min after IN administration did not differ significantly, suggesting that C_max_ likely occurred between these time points. Similarly, in the brain, the time to reach C_max_ was 5 min for F4 and 15 min for the RH solution. The elevated RH levels measured in the olfactory bulbs primarily reflect their function as a transit point along the olfactory nerve route for achieving brain targeting [34]. This observed rapid brain onset following IN administration can be explained by the direct anatomical access of the nasal cavity to the brain. Nasal formulations were effectively delivered to the intended deposition sites, enabling RH to utilize two main absorption pathways: (1) direct nose-to-brain delivery via the olfactory and trigeminal neural routes [35], and (2) systemic absorption through the rich vascular network of the respiratory epithelium [36]. The API’s physicochemical properties—particularly its molecular weight below 1 kDa—support its suitability for nasal uptake [37]. Notably, RH concentrations in the olfactory bulbs were higher than in other brain regions, suggesting preferential drug accumulation in this region following nasal administration.

### 2.5. Serum RH Non-Compartmental Pharmacokinetic Analysis

**Figure 4:**
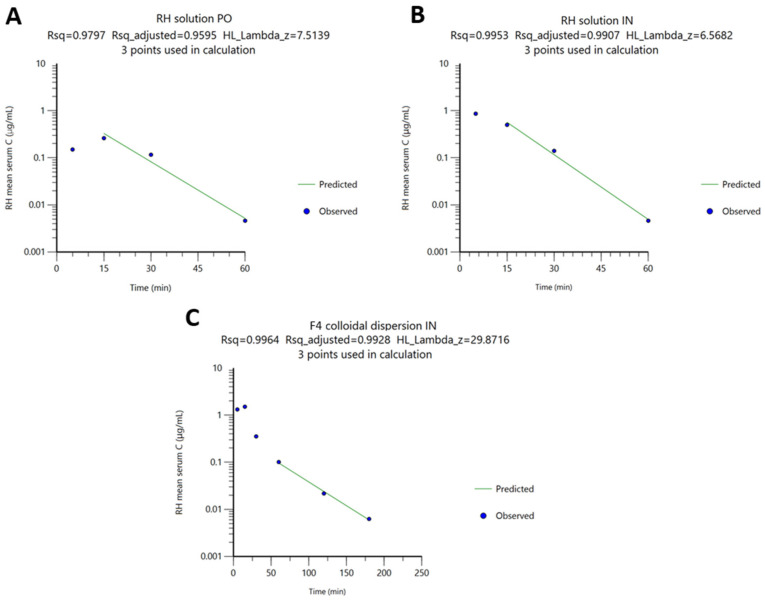
Observed (•) and predicted (green line) RH serum concentration (log scale) vs. time, after (**A**) PO administration of RH solution, (**B**) IN administration of RH solution, and (**C**) IN administration of F4 colloidal dispersion.

The serum concentration–time profiles of RH following PO administration of RH solution, IN administration of RH solution, and IN administration of the F4 colloidal dispersion are presented in Figure 2. The PK parameters derived from NCA are summarized in Table 3 and Figure 4.

The reported RH serum levels following IN administration vary considerably in the literature, primarily due to differences in formulation type, administered dose, and sampling protocols. For example, IN delivery of a radiolabeled complex of ^99m^Tc-RH in situ gel at 0.05 mg/kg in rats resulted in a C_max_ of 2.4 ± 0.8% radioactivity/g and a T_max_ of 30 ± 10 min [30], whereas administration of a 3 mg/kg dose in mice produced a C_max_ of 0.03287 ± 0.0016 μg/mL and a T_max_ of 10 ± 2.14 min [9]. Rao et al. [9] employed their work by using doses comparable to F4 (4 mg/Kg). It is noted that F4 produced a shorter T_max_ and a significantly higher C_max_ compared to their RH in situ gel. These results suggest that the formulation design, preparation method, and selected excipients of F4 positively influenced the RH serum’s bioavailability, resulting in elevated RH serum concentrations and more rapid drug absorption.

In the present study, the NCA sparse data methodology of orally administered RH solution revealed low systemic exposure (AUC_0→t_ = 7.06 ± 3.05 min·µg/mL; C_max_ = 0.26 ± 0.16 µg/mL) and rapid clearance (CL_S_ = 42.2 mL/min), consistent with limited absorption due to extensive first-pass metabolism [38,39]. This is in line with human PK studies reporting oral bioavailability of 36–57% [38,39], primarily due to cytochrome P450 1A2-mediated metabolism and the formation of inactive metabolites through N-despropylation and hydroxylation [40].

IN administration of RH solution increased both exposure (AUC_0→t_ = 15.9 ± 1.67 min·µg/mL) and peak concentration (C_max_ = 0.86 ± 0.13 µg/mL), reduced clearance (CL_S_ = 6.26 mL/min), and shortened T_max_ to 5 min. Additionally, the F_rel(Serum)_ compared with oral dosing was 674%, indicating the superiority of nasal administration compared to oral administration, offering rapid and efficient mucosal absorption. These results highlight the advantages of the IN route in overcoming the limitations associated with oral delivery [41].

The F4 colloidal dispersion administered intranasally yielded the highest systemic exposure (AUC_0→t_ = 42.7 ± 4.29 min·µg/mL; C_max_ = 1.51 ± 0.22 µg/mL), corresponding to a relative vs. oral administration bioavailability, F_rel(Serum)_, equal to 1815%, and representing a 2.7-fold increase compared to the IN RH solution. Overall, these results demonstrate that F4 significantly enhanced the nasal absorption and systemic bioavailability of RH relative to the intranasal RH solution, resulting in considerably greater exposure than the oral route. The superior performance of the F4 colloidal dispersion may be attributed to enhanced solubilization of RH, improved mucosal penetration due to nanoscale particle size, and potential interactions with mucosal surfaces that prolong residence time. As demonstrated in our previous study [11], the incorporation of RH into the hybrid polymer/surfactant/CD system significantly enhanced its in vitro release through the regenerated cellulose membranes. This improvement is likely attributed to the increased solubility of RH within the system due to the presence of the excipients. The components used for the preparation of the hybrid F4 system positively affected the RH PK profile by enhancing mucosal permeation. More specifically, the biocompatible PEO-b-PCL block copolymer, Tw80, and CDs serve as permeation enhancers by interacting with the mucosal epithelium, thereby influencing the integrity of the barrier and ultimately enhancing RH permeability [42,43,44,45,46,47]. This suggests a positive impact on drug absorption, resulting from the synergistic interactions of the three components on drug transport across the membranes, which was also proven by the ex vivo permeation studies. The ability of PEO and PCL homopolymers, and PEO–PCL copolymers, to enhance nasal mucosal permeability and drug delivery to the brain is also supported by previous in vivo studies. Quercetin-loaded PCL nanocapsules administered intranasally demonstrated superior brain delivery compared with quercetin dispersions given either orally or intranasally [45]. Similarly, IN melatonin-loaded PCL nanoparticles in rats exhibited nose-to-brain transport confirmed by fluorescence tomography and achieved higher brain AUC and targeting indices than the free drug delivered via the IN or PO routes [44]. Shah et al. [46] showed that IN administration of mPEG–PCL–Tat nanomicelles in rats resulted in approximately 2.0% injected dose per gram (% ID/g) in brain tissue—substantially exceeding the ~0.5% ID/g observed after intravenous administration of the same formulation and the ~0.2% ID/g obtained with naked siRNA—demonstrating superior mucosal penetration and brain uptake. The amphiphilic architecture of PEO–PCL copolymers combine the muco-inert and of hydrophilic nature PEO with the biodegradable, lipophilic properties of PCL, thereby improving epithelial permeation and protecting labile cargo during transit. Although further IN in vivo studies on other PEO–PCL-based systems remain limited, current findings highlight their strong potential as carriers for enhancing nasal drug absorption and achieving therapeutically relevant brain concentrations [43].

The permeation-enhancing properties of CDs in nasal delivery have also been well documented. A 1% *w*/*v* concentration of HPβCD or randomly methylated βCD enhanced melatonin permeation through human airway mucosal models by approximately 125% [42]. CDs enhances drug solubility and permeation by forming inclusion complexes and may interact with epithelial tight junctions. Meanwhile, the surfactant Tw80 has been used in nasal formulations to improve bioavailability. Specifically, in vivo studies combining Tw80 with chitosan further boosted peptide uptake in nasal models, underscoring its potential as an adjunct permeation enhancer in IN delivery systems [47].

This PK study revealed significant differences in systemic exposure among the F4 colloidal dispersion and RH solutions given orally and intranasally, highlighting the impact of both the F4 formulation and the nasal route of administration on enhancing the systemic absorption of RH. To the best of the authors’ knowledge, this is the first report in the literature comparing the PKs of RH after IN and PO administration in mice or in another animal model. Therefore, direct comparisons with previous studies employing these two routes are not currently feasible.

### 2.6. Brain RH Non-Compartmental Pharmacokinetic Analysis

**Figure 5:**
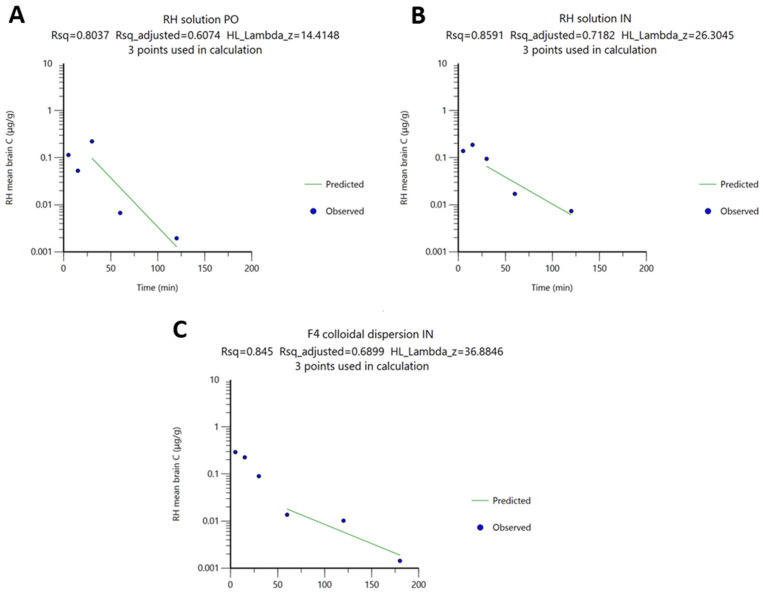
Observed (•) and predicted (green line) RH brain concentration (log scale) vs. time, after (**A**) PO administration of RH solution, (**B**) IN administration of RH solution, and (**C**) IN administration of F4 colloidal dispersion.

The brain PK parameters of RH were determined using the NCA sparse-sampling approach based on total brain concentration–time profiles. The results are summarized in Table 4 and Figure 5. Particularly, the preeminence of IN RH delivery is evident from the approximately equal (in the case of the IN RH solution) or higher (in the case of F4) AUC and C_max_ values compared to those observed following PO administration of the RH solution at a three-fold higher dose. Specifically, the brain C_max_ values were equal to 0.19 ± 0.03 μg/g (n = 5) for the IN RH solution and 0.29 ± 0.06 μg/g (n = 4) for IN F4, while the oral administration of the RH solution showed drug levels in the brain equal to 0.22 ± 0.12 μg/g (n = 5). The F4 formulation administered intranasally also produced the highest brain AUC_0→t_ value (8.26 ± 0.14 min·µg/g), exceeding both the PO RH solution (6.86 ± 2.63 min·µg/g) and IN RH solution (6.49 ± 0.69 min·µg/g), indicating enhanced brain delivery.

The time–RH concentration profiles further revealed notable differences in the uptake of drug into the brain between the F4 formulation and RH control solutions. More specifically, the NCA revealed that the T_max_ was 5 min for IN F4, 15 min for the IN administration of RH solution, and 30 min for the PO administration of the RH solution.

Previous studies also highlight variability in RH brain PKs after IN administration. Khan et al. [30] reported that IN delivery of a radiolabeled complex of ^99mTc^-RH in situ gel (0.05 mg/kg, rats) yielded a brain C_max_ of 1.80 ± 0.85% radioactivity/g with a T_max_ of 30 ± 13 min, while Rao et al. [9] observed a C_max_ of 0.06874 ± 0.0018 µg/mL and a T_max_ of 20 ± 1.6 min in mice receiving an RH in situ gel at 3 mg/kg. These discrepancies likely reflect differences in dose, formulation type, animal type, and experimental dose protocol. Notably, neither study performed perfusion prior to brain tissue collection, meaning that their reported C_max_ values may also include the amount of RH within the cerebral vasculature.

The CL_Β_ values after IN administration of the RH solution and F4 formulation were 2.9 and 3.6 times lower than that after PO administration of the RH solution. This outcome can be explained by differences in the absolute bioavailability of RH between the two administration routes. More specifically, in oral administration, CL_B_ reflects only the fraction of drug that crosses the BBB, which is significantly reduced by extensive first-pass metabolism, thereby lowering systemic RH concentrations available for brain uptake [48]. Additionally, the three times higher oral dose than that used intranasally should be considered, as clearance is calculated from the dose-to-AUC ratio. By contrast, the nose-to-brain route likely delivers RH more directly to the CNS, bypassing systemic circulation. Consequently, IN administration of the RH solution and the F4 colloidal dispersion increased brain exposure, yielding F_rel(Brain)_ values of 294% and 363%, respectively.

As demonstrated in the ex vivo permeation studies, the excipients of F4 colloidal dispersion enhanced RH permeation across the nasal mucosa by 1.7-fold compared to the pure RH solution. The studies using the rabbit nasal mucosa as a permeation barrier also showed partial retention of the drug within the tissue (12.40 ± 0.87% of the initial loading dose).

The differences in RH t_1_/_2_ values between the RH solutions and the F4 formulation, as well as between administration routes (PO and IN), are consistent with variations in the λ obtained from log-transformed brain concentration–time profiles (Figure 5). These variations likely arise from differences in sampling schedules and the time points at which concentrations approach zero. In all cases, λ was calculated by linear regression of the last three log-transformed concentration–time points (Figure 5).

Notably, F4 achieved the highest value relative to PO administration serum (F_rel(Serum)_ = 1815%) and brain bioavailability (F_rel(Brain)_ = 363%), suggesting that its superior brain exposure was driven primarily by systemic circulation. Overall, these results indicate that F4 delivers a significantly higher total amount of RH to the brain than the RH solution, making it the more effective formulation for maximizing CNS exposure.

In summary, the enhanced brain exposure observed with formulation F4 likely resulted from increased systemic absorption, as RH levels were higher in serum than in the brain, suggesting that the intranasally administered dose reached the brain through BBB penetration. Similarly, Gao et al. [49] showed that Tw80 improved the systemic absorption of tetramethylpyrazine after IN dosing, with elevated brain levels arising from higher plasma concentrations rather than direct nose-to-brain transport. Consistently, in our study, the excipients (P407, Tw80, and CDs) appeared to primarily enhance absorption into the bloodstream after IN delivery, and the increase in brain levels was largely a result of these higher plasma levels.

### 2.7. Data Analysis by Relative Bioavailability Values

According to the FDA, RH demonstrates linear PKs within the 1–8 mg dose range when administered three times daily in humans [50]. Assuming similar linearity in the present study, two parameters, namely DTE(%) and DTP(%), were applied to evaluate brain delivery following IN administration. DTE(%) measures the overall efficiency of RH brain delivery via IN vs. PO dosing, while DTP(%) estimates the proportion attributable to direct nose-to-brain transport relative to systemic absorption.

The IN administration of both the RH solution and F4 resulted in slightly positive DTE and negative DTP values, indicating that brain exposure primarily arose from systemic absorption followed by BBB transport rather than direct nose-to-brain pathways (olfactory or trigeminal). Notably, the IN RH solution showed higher brain targeting efficiency (DTE = 42%) than F4 (DTE = 20%). However, when the overall PK profile is considered, the superiority of F4 becomes evident. Despite being administered at a three-fold lower dose than the oral formulation, F4 achieved the highest brain exposure (AUC_0→t_ = 8.27 min·µg/g) and peak concentration (C_max_ = 0.29 µg/g), exceeding those of both the IN (AUC_0→t_ = 6.49 min·µg/g) and oral (AUC_0→t_ = 6.86 min·µg/g) RH solutions. Moreover, F4 produced markedly higher relative bioavailability in both serum (F_rel(Serum)_ = 1815%) and the brain (F_rel(Brain)_ = 363%) compared to oral administration, as well as a longer apparent half-life (t_1/2_ = 36.9 min) and lower clearance (CL_B_/F = 12.0 g/min), indicating sustained systemic exposure and efficient drug retention. Additionally, F4 achieved the highest brain exposure but also the most negative DTP (–363), suggesting that the elevated RH levels in the brain after IN administration of F4 were driven primarily by systemic circulation, through the BBB. Overall, F4 produced higher brain exposure than the RH solution given intranasally or orally, suggesting that although direct nose-to-brain transport is limited, the IN delivery of F4 remains a more effective formulation for enhancing CNS exposure compared to both the oral route and the simple nasal solution.

## 3. Materials and Methods

### 3.1. Chemicals and Reagents

The amphiphilic PEO-b-PCL diblock copolymers were synthesized via ring-opening polymerization of the ε-caprolactone monomer using monohydroxy-PEG as a macroinitiator [51,52,53]. PEO-b-PCL_1_, PEO-b-PCL_2_, and PEO-b-PCL_3_ block copolymers were produced, containing 15%, 30%, and 53% wt of PCL (hydrophobic component), respectively. The synthetic procedure and the molecular characteristics of the PEO-b-PCL block copolymers are reported in our previous publication [11]. Polysorbate 80 (Tw80) and HPβCD were obtained from Sigma-Aldrich Chemical Co. (St Louis, MO, USA). All systems were prepared using HPLC-grade water. Chloroform and the other reagents used were of analytical grade and purchased from Sigma-Aldrich Chemical Co. (St Louis, MO, USA). RH was kindly donated by Pharmathen (Athens, Greece). Caffeine-D9 was purchased from Fisher Scientific (Waltham, MA, USA). HPLC-grade solvents (water and methanol) and reagents were obtained from Fischer Scientific (Pittsburgh, PA, USA). Isoflurane (IsoVet^®^), used for short-term anesthesia, was acquired from Biovet (Thessaloniki, Greece).

### 3.2. Preparation of PEO-b-PCL/Tw80/βCD/RH Colloidal Dispersions

The colloidal dispersions F1–F12 (PEO-b-PCL/Tw80/MβCD or PEO-b-PCL/Tw80/HPβCD, using PEO-b-PCL polymers with an increasing proportion of the hydrophobic PCL block), were formulated following the protocol applied in our previous study [11]. In brief, the colloidal dispersions were prepared by the conventional thin-film hydration method, with a polymer/surfactant weight ratio of 70:30 *w*/*w* and the incorporation of βCD at a (polymer/surfactant)/CD weight ratio of 80:20 *w*/*w*. Further incorporation of RH was achieved at a weight ratio to a system/RH ratio of 10:1 or 10:5 (Supplementary Material, Section S1).

### 3.3. Ex Vivo Mucosal Permeation Experiments

Ex vivo permeation experiments were performed using Franz diffusion cells and rabbit nasal mucosa as a model barrier [54]. Nasal mucosa was extracted on the day of the experiment from rabbit heads collected from a local slaughterhouse (Athens, Greece). Mucosa extraction was carried out, and the barrier consisted of both the epithelial barrier and connective tissue. Following the method of Papakyriakopoulou et al. [41], the isolation of the mucosa was achieved by using surgical scissors to make incisions on each side of the septum in both nostrils. The ethmoidal air cells (ethmoid sinus structures) were removed with surgical forceps to facilitate access to the nasal septum. The mucosa used for the permeation experiments was isolated from the septal region, which is lined with respiratory epithelium. All teeth were extracted from both sides. The nasal bone was then cut vertically at the end of the diaphragm (near the eyes) with surgical scissors, and the diaphragm was removed. A spatula was used to gently isolate the mucosa from both sides of the septum, ensuring it remained hydrated with saline solution throughout the process.

After extraction, the receptor compartment of the Franz cells was filled with PBS (pH 7.4), and a magnetic stirring bar was inserted. The mucosa was mounted between the donor and receptor compartments of the Franz diffusion cell, with the mucosal side facing the donor. The donor compartment was sealed with Parafilm^®^ Sigma-Aldrich Chemical Co. (St Louis, MO, USA) during the experimental procedure.

The dose of the colloidal dispersions examined was equal to 0.05 mg. More specifically, 50 μL of the formulation with a concentration of API equal to 1 mg/mL (corresponding to a weight ratio of (polymer/Tw80/CD)/RH of 10:1 *w*/*w*) was placed in the donor compartment and diluted with 50 μL of buffer mimicking the pH of the nasal mucosa (pH 5.6). For a formulation with an API concentration of 5 mg/mL (corresponding to a weight ratio of (polymer/Tw80/CD)/RH of 10:5 *w*/*w*), 10 μL of the formulation was placed in the donor compartment and diluted with 90 μL of the buffer (pH 5.6).

An RH solution with a concentration of 0.5 mg/mL in a buffer with pH 5.6 was also prepared and tested by introducing 100 μL of the solution into the donor compartment. The donor and receptor compartments were both covered with Parafilm^®^ to prevent evaporation. All experiments lasted 2 h. At specific time intervals (15, 30, 45, 60, 75, 90, 105, and 120 min), 0.5 mL was sampled from the receptor compartment and replaced by an equal volume of fresh PBS (pH 7.4).

At the end of the experiment, to recover the RH amount accumulated in the tissue, the mucosa was comminuted with a surgical blade and then homogenized using a small pestle or an Ultra-Turrax^®^ IKA (T10 basic model, IKA^®^-Werke GmbH & Co. KG, Staufen, Germany) with 300 μL of HPLC-grade water for 30 s. The procedure was repeated three times. After that, it was further homogenized with 300 μL of HPLC-grade acetonitrile for 30 s. After homogenization, the extract was diluted and centrifuged at 10,000 rpm for 10 min before HPLC analysis. The amounts of RH recovered from the mucosa, receptor, and donor compartments were used to calculate the mass balance. All experiments were carried out at least four times.

In addition, the values of the flux across the nasal mucosa (J_NM_) barrier to the receptor compartment of the Franz cells were obtained with Equation (1). Apparent permeability (*P_app_*) was calculated by dividing flux (J_NM_) by the initial drug concentration in the donor compartment of the Franz cells (C_0_), as described by Equation (1) [55]:

(1) $$P_{app}\;=\;\frac{J}{C_{0}}$$

### 3.4. Preparation of RH Solutions and Formulation for In Vivo Pharmacokinetic Studies

#### 3.4.1. Drug Solution for Gavage Administration

The RH oral solution was prepared by dissolving a measured quantity of RH raw material in water for injection (WFI) to obtain a final concentration of 1.5 mg/mL. Each animal received 0.2 mL of the solution (theoretical dose of RH equal to 0.3 mg or 12 mg/kg) via the oral gavage technique. To ensure the stability of the active compound, the solution was freshly prepared on the day of administration.

#### 3.4.2. Drug Solution for Nasal Administration

The RH oral solution was prepared by dissolving a measured quantity of RH raw material in WFI to achieve a concentration of 5.0 mg/mL. A 20 μL volume (theoretical dose of RH equal to 0.1 mg or 4 mg/kg) was gradually administered into each animal’s nostrils by alternating drops between the nostrils. To ensure the stability of the active compound, the solution was freshly prepared on the day of administration.

#### 3.4.3. Preparation of PEO-b-PCL/Tw80/βCD/RH Colloidal Dispersion for Nasal Administration

The preparation of the selected colloidal dispersion for the implementation of in vivo PK studies was achieved following the procedure outlined in Section 3.2. For the nasal administration of the colloidal dispersions in mice, a volume of 20 μL (theoretical dose of RH equal to 0.1 mg or 4 mg/kg) was slowly instilled into the nostrils of each animal, with alternating drops between the two nostrils until the full dose was delivered. The colloidal dispersion was prepared two days prior to administration, although drug content was shown to remain stable for at least six months stored at 4 °C in an airtight container [11].

### 3.5. In Vivo Animal Experiments

#### 3.5.1. Animals and Housing Conditions

Animal studies were carried out at the Centre of Clinical, Experimental Surgery and Translational Research of the Biomedical Research Foundation of the Academy of Athens. This facility is officially licensed for both “breeding” and “experimental” use, in accordance with Greek Presidential Decree 56/2013, which harmonizes national legislation with the European Community Directive 2010/63 on the Protection of Animals used for Experimental and Other Scientific Purposes [56]. Male C57BL/6J mice were used in the study, which were maintained under specific pathogen-free conditions in individually ventilated cages (Techniplast, Varese, Italy). Environmental conditions were constant, including a 12 h light/dark cycle, a temperature of 22 ± 2 °C, and humidity maintained at 45 ± 10%. The mice were fed on irradiated pellets (4FR22CS diet, Mucedola, Milan, Italy) and had access to tap water ad libitum. Cage bedding consisted of corncob granules (REHOFIX^®^, J. Rettenmaier & Söhne, Rosenberg, Germany), and all cages and bedding were changed weekly. All mice were routinely monitored for health status under a screening program that complied with the guidelines set by the Federation of European Laboratory Animal Science Associations (FELASA). The experimental protocol of the study was approved by the Veterinary Authorities of the Region of Athens, Greece (Ethical approval num. 1198826/Date of approval: 4 October 2023). All procedures were conducted in accordance with the ARRIVE guidelines and associated guidelines, such as the EU Directive 2010/63/EU for animal experiments [56].

#### 3.5.2. In Vivo Study Dosing and Sampling Protocols

Six-week-old to eight-week-old male C57BL/6J mice were weighed (24.1 ± 1.84 g) and randomly divided into three groups, each one receiving a different treatment: (a) the PO group of RH solution (1.5 mg/mL) (30 mice) was further divided in 6 subgroups (5 animals per subgroup), each subgroup representing one sampling time point (5, 15, 30, 60, 120 and 240 min after the treatment); (b) the IN group of RH solution (5.0 mg/mL) (25 mice) was further divided in 5 subgroups (5 animals per subgroup), each subgroup representing one sampling time point (5, 15, 30, 60 and 120 min after the treatment); and (c) the IN group of the F4 colloidal dispersion (5.0 mg/mL) (25 mice) was further divided in 6 subgroups (3–5 animals per subgroup), each subgroup representing one sampling time point (5, 15, 30, 60, 120 and 180 min after the treatment).

The PO group received 0.2 mL of the 1.5 mg/mL drug solution by gavage to a theoretical dose of 0.3 mg or 12 mg/kg (mean actual dose of 12.8 ± 0.6 mg/kg). The theoretical oral dose of 12 mg/kg RH was selected based on prior rodent studies demonstrating systemic tolerability at up to 15 mg/kg/day in chronic dosing models [50,57]. For the IN groups (b and c), a dose of three times lower (theoretical dose of 0.1 mg or 4 mg/kg and mean actual dose of 3.9 ± 0.3 mg/kg and 4.3 ± 0.2 mg/kg, respectively) was selected, with the goal of achieving effective brain concentrations through the nose-to-brain delivery pathway [58,59]. The IN doses for both the RH solution and F4 formulation were derived from an interspecies dose conversion between human and mouse equivalents, referencing the marketed formulation Requip [60]. Based on this calculation, the highest permissible equivalent dose for a 25 g mouse was estimated at 4.2 mg/kg. Accordingly, a theorical IN dose of 4.0 mg/kg was selected, while the PO group received a three-fold higher dose to evaluate whether the lower IN dose could achieve greater bioavailability of RH through enhanced nose-to-brain delivery. Table 5 presents an overview of the experimental protocol for the implementation of in vivo studies, including the tested formulations, mode of administration, RH dose (mg), and both theoretical and experimental doses expressed in mg/kg.

The IN administration of both the RH solution and F4 colloidal dispersion was carried out under isoflurane anesthesia (Isovet^®^, Biovet, Thessaloniki, Greece) using a calibrated vaporizer (Abbott Laboratories, Abbot Park, IL, USA) set to a minimum alveolar concentration (MAC) of 2–4% in 100% oxygen. Anesthetic delivery was facilitated via a 3D-printed, coaxially designed rodent face mask, which ensured effective gas delivery and scavenging [61]. Throughout the procedure, animals were monitored for abnormal respiratory patterns, and a negative foot reflex was used to indicate a surgical plane of anesthesia. Mice were gently restrained by grasping the loose skin at the back of the neck, and IN administration was performed by slowly instilling 20 µL of either the RH solution or F4 formulation into both nostrils using a pipette. The full administration process lasted approximately one min. Anesthesia was maintained only for the duration necessary to complete the administration and handling. Mice recovered rapidly, within approx. 1 min, following administration and were returned to their cages, remaining conscious for the duration of the experiment.

At specific time points, blood samples were collected from the mice in the respective groups. The blood samples were taken by submandibular bleeding of mice using a lancet, transferred into non-heparinized Eppendorf^®^ tubes (Hamburg, Germany), and then centrifuged (10,000 rpm for 15 min at 4 °C) to separate the serum. Afterwards, mice were re-anesthetized with isoflurane (MAC 2–4%) and euthanized to collect brain samples. The brain samples were collected after total body perfusion [62]. Specifically, the abdominal area was sterilized with 70% ethanol (*v*/*v*) and then opened with a surgical blade. The xiphoid cartilage was lifted, and the chest opened. Then, to release the heart, any tissue connecting to it was carefully trimmed. The perfusion procedure was performed by means of a syringe pump (PLUS SEP-12S) connected with a 23G butterfly needle inserted into the heart’s left ventricle for approximately 3 min at a flow rate of 10.83 mL/min (total volume: ~32 mL) using cold phosphate buffer solution (PBS, pH 7.4). The PBS was maintained at a temperature of approximately 4 °C throughout the procedure to ensure effective removal of residual blood and the preservation of tissue integrity. Following perfusion, the brain was carefully removed and weighed. For the IN groups (b and c), the olfactory bulbs were isolated and weighed separately to allow for distinct quantification of drug levels in that region. All collected samples, including the serum, brain, and olfactory bulbs, were immediately frozen and stored at −70 °C until further processing for extraction and ultrahigh-performance liquid chromatography (UHPLC) analysis.

### 3.6. RH Extraction from Biological Samples

#### 3.6.1. RH Extraction from Serum

For RH extraction, a 25 μL serum sample was mixed with 50 μL of internal standard (ISTD) solution (caffeine-D9 0.4 μg/mL in methanol) and 25 μL of mobile phase (a 1:1 mixture of methanol and water). The presence of organic solvents caused protein precipitation, which was then separated by centrifugation at 10,000 rpm, at 4 °C for 10 min. From the resulting supernatant, 1 μL was withdrawn and injected into the UHPLC system for analysis. The same procedure was applied for the preparation of the blank serum samples spiked with RH standard solutions, used for generating the calibration curves.

#### 3.6.2. RH Extraction from Mice Brain and Olfactory Bulbs

On the day of analysis, each brain sample was homogenized using a T10 ULTRA-TURRAX^®^ (IKA Werke, Staufen im Breisgau, Germany) in WFI (tissue:WFI ratio 1:1 *w*/*w*). Each olfactory bulb was homogenized separately in a 2 mL Eppendorf^®^ microtube by manually grinding the tissue with a disposable polypropylene pestle (Sigma-Aldrich, St. Louis, MO, USA) [63]. For RH quantification, 25 μL of homogenate tissue was vortexed with 50 μL of ISTD (caffeine-D9, 0.4 μg/mL in methanol) and 25 μL of mobile phase. The mixture was centrifuged for 10 min at 10,000 rpm at 4 °C, and 1 μL of the supernatant was collected and directly injected into the UHPLC system.

### 3.7. Bioanalytical Method for Ropinirole Hydrochloride

#### 3.7.1. Preparation of Stock and Working Standard Solutions

Stock and Working Standard (WS) solutions were prepared by dissolving or diluting adequate amounts of the reference materials in MeOH and mobile phase, respectively. Two different series of stock solutions were prepared separately for calibration WS and Quality Control (QC) samples. RH stock solutions (I and II) were prepared at a concentration of 200 μg/mL by dissolving 10 mg of the reference API in methanol (50 mL). Dilutions of stock solution I were used to produce WS solutions at a concentration range from 0.04 to 1.60 μg/ mL, while dilutions of stock solution II were made to provide QC solutions at three different concentrations (0.04 μg/mL, 0.40 μg/mL, and 1.60 μg/mL) to investigate intra- and inter-run variations. Stock solutions were stored at −26 °C in vials, while WS solutions were prepared on the day of analysis. A stock solution of ISTD (caffeine-D9, 1 mg/mL) in methanol was further diluted with the same solvent, at a final concentration of 0.40 μg/mL. Matrix calibration curves and QC samples were prepared daily in blank mouse plasma and brains, spiked with 25 μL of the WS or QC solutions and 50 μL of the ISTD solution.

#### 3.7.2. UHPLC-TIMS-QTOF-MS Methodology for RH Quantification in Biological Samples

##### Analysis of Biological Samples by UHPLC-TIMS-QTOF-MS

RH concentrations in serum and brain tissue samples were quantified using a validated UHPLC-TIMS-QTOF-MS method [64]. In brief, caffeine-D9 was selected as the internal standard based on its suitability and consistent analytical performance within the developed method. The assay has been fully validated, demonstrating acceptable linearity over the range of 0.010–0.300 μg/mL (R^2^ = 0.9997 for serum and 0.9993 for the brain). Precision and accuracy were within acceptable limits, with within-run accuracy (Er, %) values below 6.7% for low, medium, and high QC samples. Between-run accuracy ranged from −11.1% to −13.7%, recovery [98.7% ± 1.57%, RSD (%) for serum and 97.3% ± 4.62%, RSD (%) for brain], limit of detection (LOD = 0.0031 ± 0.0004 μg/mL), and lower limit of quantification (LLOQ = 0.0092 ± 0.0124 μg/mL for serum and 0.0094 ± 0.0006 μg/mL for the brain), as described in detail in our previous work [64].

Specifically, the quantification of the biological samples was carried out by using an UHPLC system (Elute LC series, Bruker Daltonics, Bremen, Germany) coupled to a hybrid trapped ion mobility spectrometry–quadrupole time-of-flight mass spectrometry system (TIMS-QTOF) (timsTOF pro 2, Bruker Daltonics, Bremen, Germany).

Chromatographic separation was performed on an Elute (Bruker Daltonics, Bremen, Germany) UHPLC equipped with a Solo C_18_ column (2.1 × 100 mm, 1.8 μm) (Bruker, Daltonics), thermostated at 40 °C. In positive ionization mode, the mobile phases consisted of water/methanol 90:10 *v*/*v* (solvent A) and methanol (solvent B), both containing 5 mM ammonium formate and 0.01% formic acid. In negative ionization mode, the mobile phases consisted of water/methanol 90:10 *v*/*v* (solvent A) and methanol (solvent B), both containing 5 mM of ammonium acetate. A gradient elution program was applied in both ionization modes, starting at 1% B (flow rate of 0.2 mL/min) for 1 min, increasing to 39% over 2 min and then to 99.9% (flow rate of 0.4 mL/min) over the next 11 min. Solvent B was then held constant for 2 min (flow rate of 0.48 mL/min), followed by re-equilibration of the column to initial conditions for 3 min. The injection volume was set to 1 μL.

The TIMS-QTOF-MS system (timsTOF pro2, Bruker Daltonics) was equipped with a Vacuum-Insulated Probe-Heated ElectroSpray Ionization (VIP-HESI) source with the following operating conditions: end plate offset—500 V; capillary voltage—2500 V; nebulizer pressure—3.0 bar; dry gas flow—10.0 L/min; dry gas temperature—220 °C; sheath gas temperature—450 °C; and sheath gas flow—4.0 L/min. Data was acquired using bbCID (broadband Collision-Induced Dissociation) mode with optimized mass and mobility transfer parameters to enhance the coverage of small ions in the *m*/*z* range of 20–1300 and 1/K_0_ range of 0.10–1.50 V∙s/cm^2^, and with ramp time set at 150 ms. A QTOF external calibration was performed daily with the manufacturer’s solution (sodium formate clusters) covering the whole mass range of interest.

Regarding TIMS, accumulation time was dynamic, considering the loading of ions into the TIMS device through the Ion Charge Control (ICC) parameter. ICC constantly measures the signal intensity and adjusts the accumulation time automatically, increasing the dynamic range. TIMS dimension was calibrated linearly using three selected ions from the ESI Low-Concentration Tuning Mix (Agilent Technologies, Santa Clara, CA, USA) in positive mode [*m*/*z*, 1/k_0_: (118.1545, 0.5446 vs. cm^−2^), (322.048121, 0.7363 vs. cm^−2^), (622.028960, 0.9915 vs. cm^−2^), and (922.009799, 1.198 vs. cm^−2^)].

##### Identification Strategy and Quantification Procedure

A target screening approach was employed for the identification of ropinirole, using analytical evidence established from a reference standard. Data processing was conducted using Data Analysis 6.1 software (Bruker Daltonics, Bremen, Germany). Initially, the raw data files of the reference standard solution underwent internal mass calibration to minimize potential mass errors. The characteristic retention time (R_t_) for RH was determined by generating its extracted ion chromatogram (EIC) with a narrow mass window of ±5 mDa. Furthermore, the isotopic pattern was evaluated, and the IMS dimension was characterized by determining the specific reduced inversed ion mobility (1/K_0_) and the corresponding Collision Cross Section (CCS) value. Both MS and MS/MS spectra were processed to define the precursor and qualifier ion characteristics of the RH. This procedure allowed for the collection of R_t_, CCS and fragmentation data, providing robust analytical evidence for the subsequent analysis of biological samples.

The screening of mouse serum and brain samples was performed based on the evidence derived from reference standard, ensuring the successful identification of RH with high confidence. For an identification to be considered successful, the following criteria were applied: a maximum retention time shift (ΔR_t_) of 0.2 min, a mass accuracy of <5 mDa, an isotopic pattern fitting (mSigma) of <200 and a CCS shift of <2%. Additionally, the identification was confirmed by the detection of at least two characteristic qualifier ions unique to RH. For the quantification procedure, the linear signal response of the instrument was established using ropinirole reference standards across the examined concentration range. To ensure analytical reliability, a stable isotopically labeled internal standard (caffeine-D9) was introduced prior to analysis to mitigate potential ion suppression and matrix effects, which are common in complex biological tissues. Consequently, quantification was performed using relative areas, calculated by dividing the absolute peak area of ropinirole by the area of the internal standard.

### 3.8. Non-Compartmental Analysis

Sparse-sampling non-compartmental analysis (NCA) was performed for in vivo data using Phoenix^®^ 8.3.5 (Certara, Princeton, NJ, USA) to estimate PK parameters in serum, brain, and olfactory tissues. These parameters included AUC_0→t_ (the area under the concentration–time curve from time 0 to the last time point of the study), AUC_inf_ (the area under the concentration–time curve extrapolated to infinity), C_max_ (maximum observed concentration), and T_max_ (the time when C_max_ is observed). Moreover, the relative oral bioavailability of RH following the IN administration of the RH solution or F4 colloidal dispersion was calculated. The mean concentration vs. time curve was calculated and used in conjunction with the available subject information to calculate PK parameters and their standard errors (SEs). The area under the concentration–time curve (AUC_inf_) was calculated according to the log-linear trapezoidal method with extrapolation to infinity by dividing the last concentration by the terminal slope, λ. The terminal slope was estimated by linear regression analysis on the last three points of the log-transformed concentration vs. time plot. The relative bioavailability (F_rel_, %) of RH in the serum and brain following IN administration, compared to PO administration, was calculated by comparing the AUC_inf_ values according to Equation (2):

(2) $$F_{\mathrm{rel}}=\;\frac{{\mathrm{AUC}}_{\inf(\mathrm{IN})}\;\times \;{\mathrm{Dose}}_{\left(\mathrm{PO}\right)}}{{\mathrm{AUC}}_{\inf(\mathrm{PO})}\;\times \;{\mathrm{Dose}}_{\left(\mathrm{IN}\right)}}\times 100$$

where AUC_inf(IN)_ and AUC_inf(PO)_ represent the area under the concentration vs. time curve from 0 extrapolated to infinity, after IN and PO administration, respectively. Dose_(IN)_ and Dose_(PO)_ are the respective administered doses. The elimination half-life, t_1/2_, was determined as t_1/2_ = 0.693/λ, where λ is the terminal elimination rate constant. The plasma and brain clearance, CL_S_ and CL_B_, were calculated as CL_S_ = Dose/AUC_inf_ and CL_B_ = Dose/AUC_inf_, respectively, and both were adjusted by a factor of 1/F, with F representing the absolute bioavailability.

### 3.9. Relative Drug Targeting Efficiency Percentage and Nose-to-Brain Direct Transport Percentage Indexes

The relative Drug Targeting Efficiency Percentage (DTE) and nose-to-brain Direct Transport Percentage (DTP) indexes were applied to evaluate direct RH transport to the brain.

More specifically, the DTE is used to express the relative exposure of the brain to the drug following IN administration vs. oral administration according to Equation (3).

(3) $$\mathrm{DTE}=\frac{\left(\frac{{\mathrm{AUC}}_{0\to t\;(\mathrm{Brain})}}{{\mathrm{AUC}}_{0\to t\;(\mathrm{Serum})}}\right)\mathrm{IN}}{\left(\frac{{\mathrm{AUC}}_{0\to t\;(\mathrm{Brain})}}{{\mathrm{AUC}}_{0\to t\;(\mathrm{Serum})}}\right)\mathrm{PO}}\times 100$$

The DTE values range from −∞ to +∞ and values higher than 100 indicate more efficient drug delivery to the brain after IN administration in comparison to oral administration.

The DTP expresses the percentage of the dose that reaches the brain via direct routes (i.e., via trigeminal or olfactory nerves), vs. the overall brain delivery of the studied drug/compound. The following equation is used to calculate the DTP (Equation (4)):

(4) $$\mathrm{DTP}=\frac{[{\mathrm{AUC}}_{0\to t\;(\mathrm{Brain})}]\mathrm{IN}-\left[{\mathrm{AUC}}_{0\to t\;(x)}\right]}{[{\mathrm{AUC}}_{0\to t\;(\mathrm{Brain})}]\mathrm{IN}}\times 100$$

where *AUC*0 → *t* (*x*) is calculated using Equation (5):

(5) $${\mathrm{AUC}}_{0\to t\;(x)}=\frac{[{\mathrm{AUC}}_{0\to t\;(\mathrm{Brain})}]\mathrm{PO}}{[{\mathrm{AUC}}_{0\to t\;(\mathrm{Serum})}]\mathrm{PO}}\times [{\mathrm{AUC}}_{0\to t\;(\mathrm{Serum})}](\mathrm{IN})$$

The DTP values range from −∞ to 100, while any value ≤ 0 implies that the drug is primarily delivered to the brain through the systemic circulation after crossing the BBB and not directly through nose-to-brain delivery [13,64]. Oral administration was used as a reference route of administration since RH is currently administered via this route in clinical practice. The main objective was to assess whether IN administration could offer a pharmacokinetic advantage compared to conventional oral administration.

## 4. Conclusions

This study aimed to evaluate ex vivo permeation through rabbit nasal mucosa and determine the PK parameters of RH, when administered intranasally as a colloidal dispersion, compared with oral and intranasal RH solutions in C57BL/6J mice.

Ex vivo permeation studies revealed a significant increase in RH permeation across the nasal mucosa from all tested formulations compared with the pure RH solution, indicating a synergistic effect resulting from the combined action of the formulation components. Among these, formulations containing HPβCD demonstrated superior permeation enhancement than those containing MβCD. Formulation F4 was selected for further evaluation. The in vivo PK study demonstrated that IN delivery provided superior PK performance compared to conventional oral dosing. The PK parameters derived using the NCA sparse data methodology illustrated that F4 colloidal dispersion significantly enhanced both systemic and brain exposure of RH, achieving higher serum AUC and C_max_ values and reduced CL. Compared to *per os* administration, IN delivery of F4 achieved markedly higher drug concentrations in serum and brain tissue. Notably, these enhancements in F_rel_ for brain and serum were attained with a 3-fold lower administered dose.

F4 showed high systemic (F_rel(Serum)_ = 1815%) and brain (F_rel(Brain)_ = 363%) values relative to the PO administration bioavailability, underscoring its potential as an IN delivery system for efficient CNS targeting. This profile can be attributed not only to the advantages of the nasal route of administration compared to the oral one, such as circumventing first-pass metabolism, but also to the inclusion of functional excipients (PEO-b-PCL block copolymer, Tw80, and CDs) that enhance mucosal penetration and facilitate RH transport into the CNS. When directly compared to the simple intranasal RH solution, F4 demonstrated superior PK performance, yielding higher AUC and C_max_ values in both serum and brain, prolonged half-life, and reduced clearance. These improvements indicate that the colloidal matrix of F4 provided enhanced retention and absorption across the nasal mucosa, resulting in more efficient systemic uptake and, consequently, greater CNS exposure.

The novelty of this work resides in the development and application of a biocompatible hybrid colloidal system composed of PEO-b-PCL, HPβCD, and Tw80 for the IN delivery of RH. To the best of our knowledge, this is the first report employing such a hybrid system for RH nasal administration. This approach enables the use of significantly lower drug doses while achieving enhanced systemic and brain bioavailability. Importantly, this formulation enables a significant enhancement in both systemic and brain bioavailability, allowing the achievement of higher exposure at substantially lower administered doses. This dose-sparing effect represents a key advantage, potentially reducing systemic side effects and improving the safety profile of the compound. Furthermore, the proposed formulation demonstrates superior brain targeting efficiency compared to the RH solution, whether delivered orally or intranasally.

Overall, this study highlights the potential of IN RH as a non-invasive and viable alternative to oral delivery for PAD management. From a future clinical perspective, research should further assess long-term safety, therapeutic effectiveness, and the translational applicability of this approach in humans.

## Figures and Tables

**Table 1:** Composition of all the prepared PEO-b-PCL-based formulations.

PΕO-b-PCL/Tw80/CD/RH Systems		
Formulations		Weight Ratio (*w*/*w*)
F1	(PEO-b-PCL_1_/Tw80/MβCD)/RH	10:1
F2	(PEO-b-PCL_1_/Tw80/MβCD)/RH	10:5
F3	(PEO-b-PCL_1_/Tw80/HPβCD)/RH	10:1
F4	(PEO-b-PCL_1_/Tw80/HPβCD)/RH	10:5
F5	(PEO-b-PCL_2_/Tw80/MβCD)/RH	10:1
F6	(PEO-b-PCL_2_/Tw80/MβCD)/RH	10:5
F7	(PEO-b-PCL_2_/Tw80/HPβCD)/RH	10:1
F8	(PEO-b-PCL_2_/Tw80/HPβCD)/RH	10:5
F9	(PEO-b-PCL_3_/Tw80/MβCD)/RH	10:1
F10	(PEO-b-PCL_3_/Tw80/MβCD)/RH	10:5
F11	(PEO-b-PCL_3_/Tw80/HPβCD)/RH	10:1
F12	(PEO-b-PCL_3_/Tw80/HPβCD)/RH	10:5

**Table 2:** Percentage (%) of RH loading dose permeated for the tested formulations (F1–F12) across the nasal mucosa barrier (mean ± SE, n = 4) at different time points (15, 45 and 120 min).

Formulation	Permeated RH (% Loading Dose)		
	15 min	45 min	120 min
F1	1.72 ± 0.13	13.69 ± 2.41	41.15 ± 2.86
F2	1.58 ± 0.39	10.12 ± 0.63	32.05 ± 1.93
F3	2.72 ± 0.49	12.60 ± 1.88	42.22 ± 2.50
F4	2.58 ± 0.63	12.61 ± 0.97	33.40 ± 0.18
F5	0.82 ± 0.19	7.76 ± 1.33	28.16 ± 2.88
F6	1.97 ± 0.21	10.63 ± 0.29	30.91 ± 0.16
F7	3.08 ± 0.66	15.23 ± 1.67	41.07 ± 2.78
F8	3.27 ± 0.55	14.10 ± 1.96	36.11 ± 2.46
F9	2.51 ± 0.69	15.13 ± 2.01	41.41 ± 2.50
F10	4.60 ± 1.28	17.32 ± 2.86	40.21 ± 3.16
F11	2.30 ± 0.54	15.54 ± 1.66	43.38 ± 3.34
F12	3.60 ± 0.96	15.58 ± 1.67	38.56 ± 3.52
RH solution 0.5 mg/mL	0.72 ± 0.19	4.81 ± 0.52	19.62 ± 0.53

**Table 3:** Non-compartmental analysis on serum data using sparse data methodology.

PK Parameters	Estimate (% RSE)		
	RH Solution PO	RH Solution IN	F4 IN
AUC_0→t_ (min·μg)/mL	7.06 (3.05)	15.9 (1.67)	42.7 (4.29)
C_max_ (μg/mL)	0.26 (0.16)	0.86 (0.13)	1.51 (0.22)
AUC_inf_ (min·μg)/mL	7.11	16.0	43.0
T_max_ (min)	15	5	15
t_1/2_ (min)	7.51	6.57	29.9
CL_S_ (mL/min)	42.2	6.26	2.33
F_rel(Serum)_ (%)	–	674	1815

Ropinirole hydrochloride (RH), area under the curve (AUC), and clearance from serum (CL_S_) calculated as Dose/AUC and is scaled by 1/F, where F is RH serum absolute bioavailability.

**Table 4:** Non-compartmental analysis on brain using sparse data methodology and nose-to-brain delivery indexes of RH formulations.

PK Parameters	Estimate (% RSE)		
	RH Solution PO	RH Solution IN	F4 IN
AUC_0→t_ (min·μg)/mL	6.86 (2.63)	6.49 (0.69)	8.27 (0.14)
C_max_ (μg/mL)	0.22 (0.12)	0.19 (0.03)	0.29 (0.06)
AUC_inf_ (min·μg)/mL	6.90	6.63	8.35
T_max_ (min)	30	15	5
t_1/2_ (min)	14.4	26.3	36.9
CL_B_ (mL/min)	43.5	14.9	12.0
F_rel(Brain)_ (%)	-	294	363
DTE (%)	–	42	20
DTP (%)	–	−139	−402

Ropinirole hydrochloride (RH), area under the curve (AUC), and clearance from brain (CL_B_) calculated as Dose/AUC and is scaled by 1/F, where F is RH brain bioavailability.

**Table 5:** Overview of the experimental protocol for the implementation of in vivo studies, including tested formulations, mode of administration, RH dose (mg), and both theoretical and experimental doses expressed in mg/kg.

Group	Formulation	Mode of Administration	Dose (mg)	Theoretical Dose (mg/kg) in 25 g of Mouse	Experimental Dose ± SD (mg/kg)
a	RH solution, 1.5 mg/mL	PO	0.3	12	12.8 ± 0.6
b	RH solution, 5.0 mg/mL	IN	0.1	4	3.9 ± 0.3
c	F4	IN	0.1	4	4.3 ± 0.2

## Data Availability

Dataset available on request from the authors.

## Supplementary Materials

The following supporting information can be downloaded at: https://www.mdpi.com/article/10.3390/molecules31091405/s1, Figure S1. Permeation profiles of the colloidal dispersion of RH-loaded PEO-b-PCL_1_ hybrid systems (F1–F4) at a dose of 0.05 mg through rabbit nasal tissue compared to a reference RH solution (0.5 mg/mL), expressed as (**A**) quantity permeated per unit area (mean ± SE, n = 4) and (**B**) % loading dose permeated for the tested formulation (mean ± SE, n = 4). Figure S2. Permeation profiles of the colloidal dispersion of RH-loaded PEO-b-PCL_2_ (F5–F8) hybrid systems at the dose of 0.05 mg through rabbit nasal tissue compared to a reference RH solution (0.5 mg/mL), expressed as (**A**) quantity permeated per unit area (mean ± SE, n = 4) and (**B**) % loading dose permeated for the tested formulation (mean ± SE, n = 4). Figure S3. Permeation profiles of the colloidal dispersion of RH-loaded PEO-b-PCL_3_ hybrid systems (F9–F12) at the dose of 0.05 mg through rabbit nasal tissue compared to a reference RH solution (0.5 mg/mL), expressed as (**A**) quantity permeated per unit area (mean ± SE, n = 4) and (**B**) % loading dose permeated for the tested formulation (mean ± SE, n = 4). Figure S4. Ropinirole hydrochloride identification. (**a**) EIC of RH with ΔR_t_ = 0.02 min, (**b**) EIM of RH with ΔCCS = 0.12%, (**c**) MS spectrum filtered based on the R_t_ and 1/K_0_ of RH (mass error = 0 mDa and isotopic fidelity of 6.9 mSigma), and (**d**) MS/MS spectrum filtered based on the Rt and 1/K_0_ of RH (all qualifiers were detected). Table S1: Mass balance (%) of RH in each formulation (mean ± SE, n = 4); membrane retention data of each formulation expressed as the percentage (%) of the loading dose retained by the nasal mucosa barrier (mean ± SE, n = 4) and flux (*J_NM_*) (mean ± SΕ, n = 4) and the apparent permeability (*P_app_*) across the nasal mucosa barrier of the prepared formulations and RH solution (0.5 mg/mL, pH = 5.6). R-square of regression analysis of the amount of the drug permeated per unit area vs. time, across the nasal mucosa barrier is included in table [R^2^_(*NM*)_].

## Abbreviations

The following abbreviations are used in this manuscript:

APIs	other active pharmaceutical ingredients
AUC	area under the concentration–time curve
BBB	blood–brain barrier
bbCID	broadband collision-induced dissociation
CDs	cyclodextrins
CL	clearance
CL/F or CL_0_	clearance over bioavailability
C_max_	maximum drug concentration
CNS	central nervous system
DTE	drug targeting efficiency percentage
DTP	direct transport percentage
EIC	extracted ion chromatogram
EMA	European Medical Agency
f(κa)	Henry’s function
FA-PEL-CD	folate-poly(ethylene glycol)-poly(D, L-lactide)-β-CD
FBS	fetal bovine serum
FELASA	Federation of European Laboratory Animal Science Associations
F_rel_	relative bioavailability
HPβCD	hydroxy-propyl-β-CD
HPLC	high-performance liquid chromatography
ICC	ion charge control
ISTD	internal standard
LLOQ	lower limit of quantification
LOD	limit of detection
log	logarithm
MAC	minimum alveolar concentration
MβCD	methyl-β-CD
MLMPs	microparticles of mannitol/lecithin
MS	mass spectrometry
MW	molecular weight
NCA	non-compartmental analysis techniques
NOAEL	no observed adverse effect level
PAD	Parkinson’s disease
PCL	poly-ε-caprolactone
PEO	polyethylene oxide
PEO-b-PCL	poly(ethylene-oxide)-b-poly(ε-caprolactone)
PK	pharmacokinetic
PO	*per os*
QC	quality control
QTOF	quadrupole time-of-flight
PBS	phosphate buffer solution
RH	ropinirole hydrochloride
R_t_	retention time
SE	standard error
t_1/2_	elimination half-life
TIMS	trapped ion mobility spectrometry
T_max_	time to reach C_max_
Tw80	Tween 80
UHPLC	ultrahigh-performance liquid chromatography
US-FDA	U.S. Food and Drug Administration
VIP-HESI	vacuum insulated probe-heated electrospray ionization
WFI	water for injection
WS	working standard
λ	slope
