# Supplementary material for: Ex Vivo Characterization and In Vivo Nasal Delivery of Ropinirole-Loaded PEO-b-PCL/Tween 80/β-Cyclodextrin Systems in C57BL/6J Mice
Source: Molecules. 2026 Apr 23;31(9):1405. doi: 10.3390/molecules31091405 (PMC13165381; doi:10.3390/molecules31091405)
Supplement: Supplementary file 1 [file molecules-31-01405-s001.zip › molecules-4240882-supplementary.pdf]

SUPPLMENTARY MATERIAL

Ex Vivo Characterization and *In Vivo* Nasal Delivery of Ropinirole-Loaded PEO-b-PCL/Tween 80/β-Cyclodextrin Systems in C57BL/6J Mice

Elmina-Marina Saitani[1], Paraskevi Papakyriakopoulou[1], Evangelos Balafas[2], Dimitrios Damalas[3], Nikolaos Kostomitsopoulos[2], Stergios Pispas[4], Natassa Pippa[1], Nikolaos Thomaidis[3], Georgia Valsami[1,*]

1 Section of Pharmaceutical Technology, Department of Pharmacy, School of Health Sciences, National and Kapodistrian University of Athens, Panepistimiopolis Zografou, 15771 Athens, Greece; esaitani@pharm.uoa.gr; ppapakyr@pharm.uoa.gr; natpippa@pharm.uoa.gr; valsami@pharm.uoa.gr
2 Laboratory Animal Facility, Centre of Clinical and Experimental Surgery and Translational Research, Biomedical Research Foundation, Academy of Athens, 11527, Athens, Greece; vbalafas@bioacademy.gr; nkostom@bioacademy.gr
3 Laboratory of Analytical Chemistry, Department of Chemistry, National and Kapodistrian University of Athens, Panepistimioupolis Zografou, 15771, Athens, Greece. dimdamalas@chem.uoa.gr; ntho@chem.uoa.gr
4 Theoretical and Physical Chemistry Institute, National Hellenic Research Foundation, 48 Vassileos Constantinou Avenue, 11635 Athens, Greece; pispas@eie.gr

* Correspondence: valsami@pharm.uoa.gr

S1. Preparation protocol of PEO-b-PCL/Tw80/βCD/RH colloidal dispersions

The colloidal dispersions F1-F12 (PEO-b-PCL/Tw80/MβCD or PEO-b-PCL/Tw80/HPβCD, using PEO-b-PCL polymers with an increasing proportion of the hydrophobic PCL block), were prepared by the conventional thin-film hydration method. The PEO-b-PCL/Tw80 weight ratio was 70:30 *w/w*, while the subsequent incorporation of βCD was carried out at a (PEO-b-PCL/Tw80)/βCD weight ratio of 80:20 *w/w*. To prepare the system, the appropriate amount of polymer (PEO-b-PCL_1, PEO-b-PCL_2 or PEO-b-PCL_3), together with the chloroform stock solutions of Tw80 and RH (in volumes corresponding to a system/RH ratio of 10:1 or 10:5) were added to a dry round-bottom flask. Following solvent evaporation under vacuum at 40 °C using a rotary evaporator (Laborota 4000, Heidolph), a thin film was formed on the inner wall of the flask. Subsequently, an aqueous CD solution was added to the flask in a weight ratio of (polymer/Tw80)/CD equal to 80:20 *w/w*. The appropriate volume of water was added to reach a final colloidal concentration of 10 mg/mL. The hydration was carried out by slow rotation of the flask in a water bath at 40 °C. The resultant sample was subjected to two 3 min sonication cycles (amplitude 70%, cycle 0.5 s) with a 3 min pause between cycles using a probe sonicator (Bandelin Sonoplus Ultrasonic Homogenizers GM3200, 200 Watt). The quantification of RH in colloidal dispersion was performed according to a previously published HPLC-PDA [11].

Table S1. Mass balance (%) of RH in each formulation (mean ± SE, n = 4); membrane retention data of each formulation expressed as the percentage (%) of the loading dose retained by the nasal mucosa barrier (mean ± SE, n = 4) and flux (J_{NM}) (mean ± SE, n = 4) and the apparent permeability (P_{app}) across the nasal mucosa barrier of the prepared formulations and RH solution (0.5 mg/mL, pH = 5.6). R-square of regression analysis of the amount of the drug permeated per unit area vs. time, across the nasal mucosa barrier is included in table [$R^2_{(NM)}$].

Formulation	Mass Balance (%)	% of RH Dose Retained by the Nasal Mucosa Barrier	J_{NM} (µg/cm²/min)	$R^2_{(NM)}$	P_{app} (cm/min)
F1	92.54 ± 2.44	15.58 ± 1.49	$2.9\times10^{-4} \pm 0.9\times10^{-5}$	0.9930 ± 0.0011	0.57
F2	84.18 ± 1.76	14.52 ± 1.06	$2.3\times10^{-4} \pm 0.7\times10^{-5}$	0.9928 ± 0.0009	0.47
F3	95.52 ± 0.79	15.06 ± 1.47	$2.9\times10^{-4} \pm 1.0\times10^{-5}$	0.9919 ± 0.0012	0.58
F4	81.12 ± 1.23	12.40 ± 0.87	$2.5\times10^{-4} \pm 0.9\times10^{-5}$	0.9906 ± 0.0011	0.51
F5	84.36 ± 0.55	17.12 ± 1.14	$1.9\times10^{-4} \pm 0.8\times10^{-5}$	0.9886 ± 0.0009	0.38
F6	81.06 ± 0.48	14.82 ± 1.62	$2.3\times10^{-4} \pm 0.5\times10^{-5}$	0.9961 ± 0.0006	0.47
F7	87.15 ± 1.59	14.63 ± 1.36	$2.8\times10^{-4} \pm 0.7\times10^{-5}$	0.9960 ± 0.0008	0.55
F8	80.98 ± 2.57	12.35 ± 2.23	$2.3\times10^{-4} \pm 0.6\times10^{-5}$	0.9956 ± 0.0007	0.45
F9	76.57 ± 1.32	2.95 ± 0.10	$2.8\times10^{-4} \pm 0.7\times10^{-5}$	0.9954 ± 0.0008	0.55
F10	85.74 ± 1.59	11.50 ± 0.66	$2.4\times10^{-4} \pm 1.5\times10^{-5}$	0.9745 ± 0.0017	0.47
F11	76.65 ± 1.31	3.35 ± 0.34	$2.7\times10^{-4} \pm 1.3\times10^{-5}$	0.9852 ± 0.0015	0.55
F12	88.08 ± 0.38	13.24 ± 1.38	$2.5\times10^{-4} \pm 0.6\times10^{-5}$	0.9959 ± 0.0007	0.51
RH solution 0.5 mg/mL	79.90 ± 0.97	18.10 ± 0.34	$1.4\times10^{-4} \pm 0.8\times10^{-5}$	0.9769 ± 0.0009	0.28

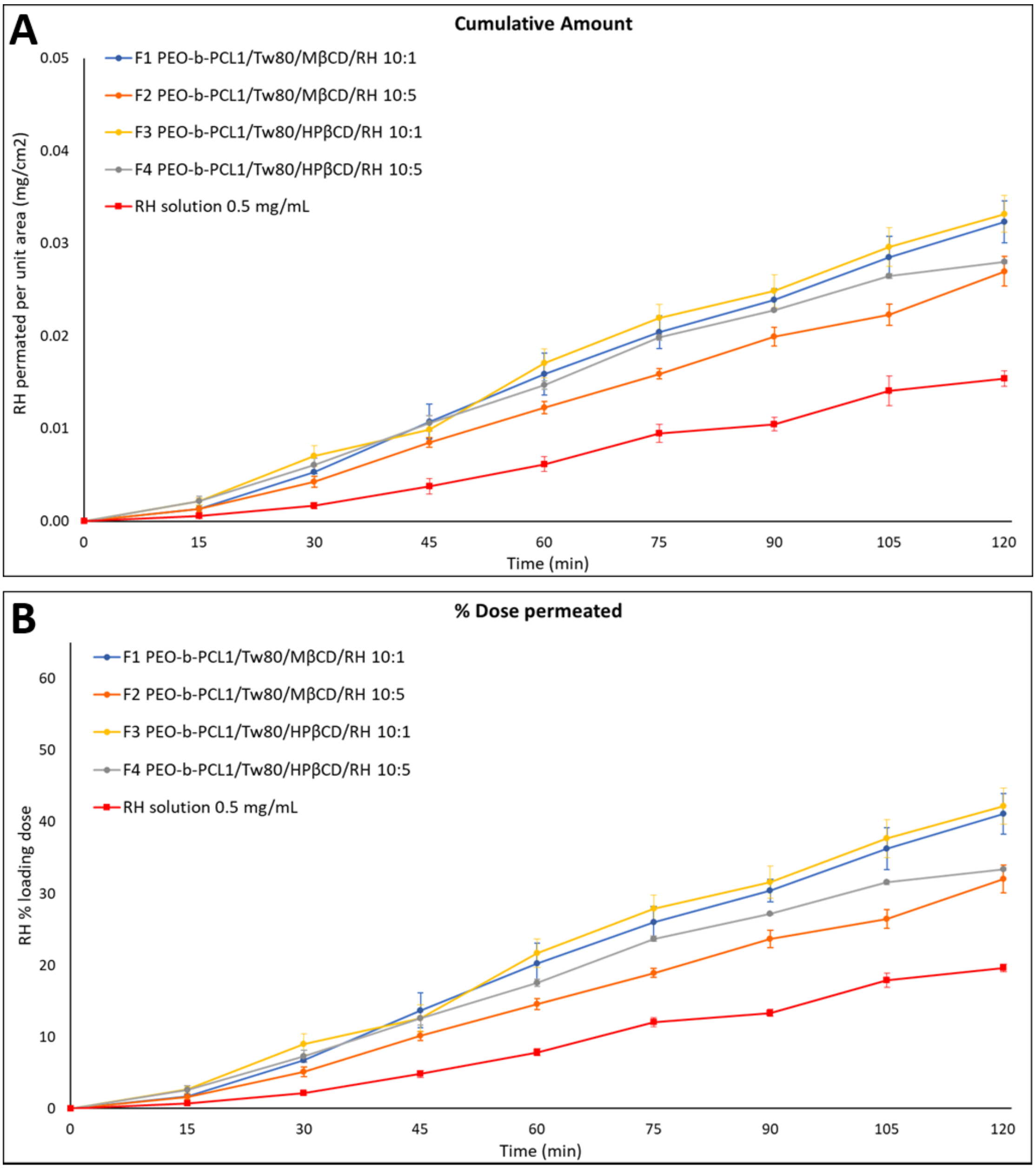

Figure S1. Permeation profiles of the colloidal dispersion of RH-loaded PEO-b-PCL_1 hybrid systems (F1–F4) at the dose of 0.05 mg through rabbit nasal tissue compared to a reference RH solution (0.5 mg/mL), expressed as (A) quantity permeated per unit area (mean ± SE, n = 4) and (B) % loading dose permeated for the tested formulation (mean ± SE, n = 4).

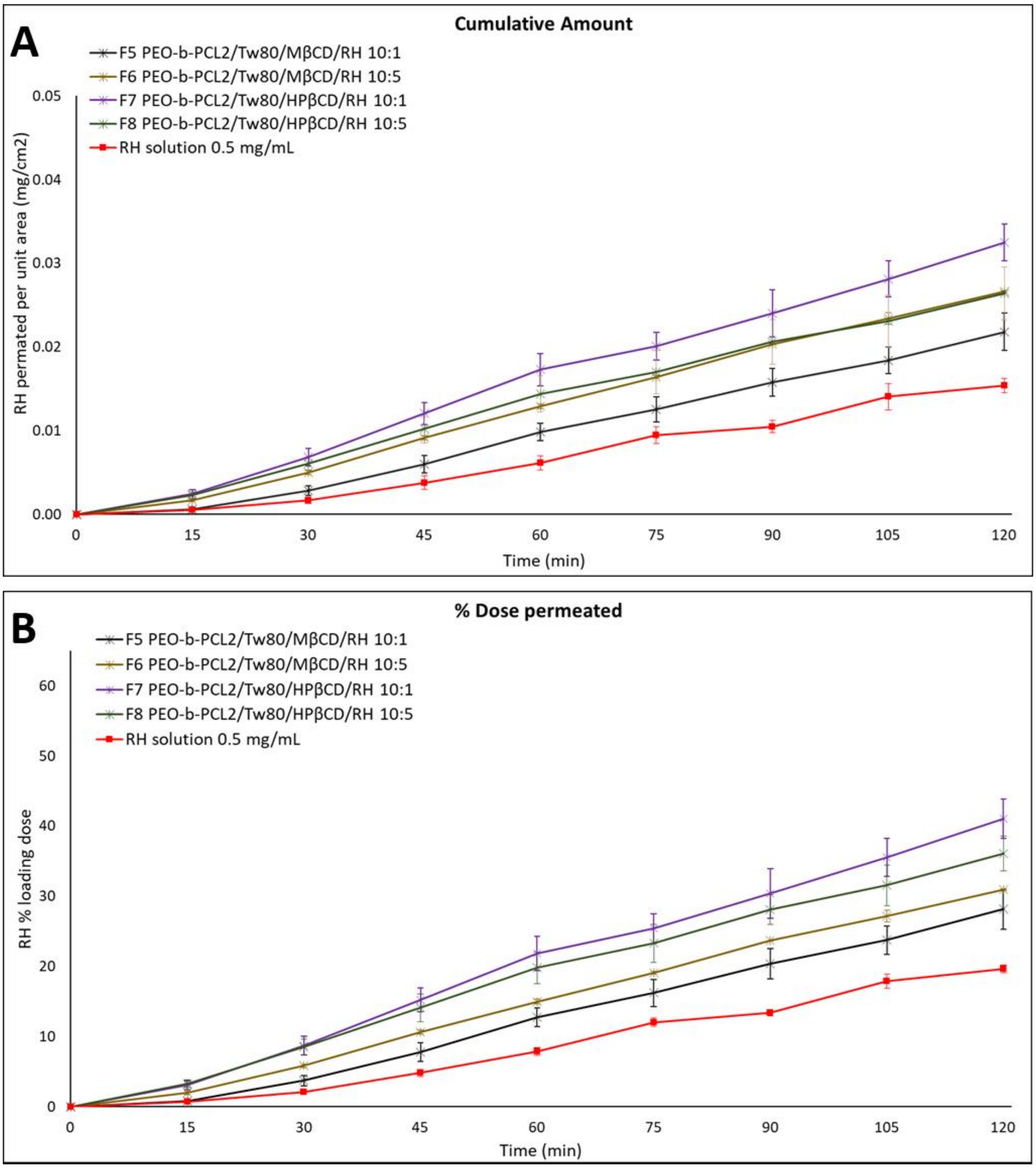

Figure S2. Permeation profiles of the colloidal dispersion of RH-loaded PEO-b-PCL_2 (F5–F8) hybrid systems at the dose of 0.05 mg through rabbit nasal tissue compared to a reference RH solution (0.5 mg/mL), expressed as (A) quantity permeated per unit area (mean ± SE, n = 4) and (B) % loading dose permeated for the tested formulation (mean ± SE, n = 4).

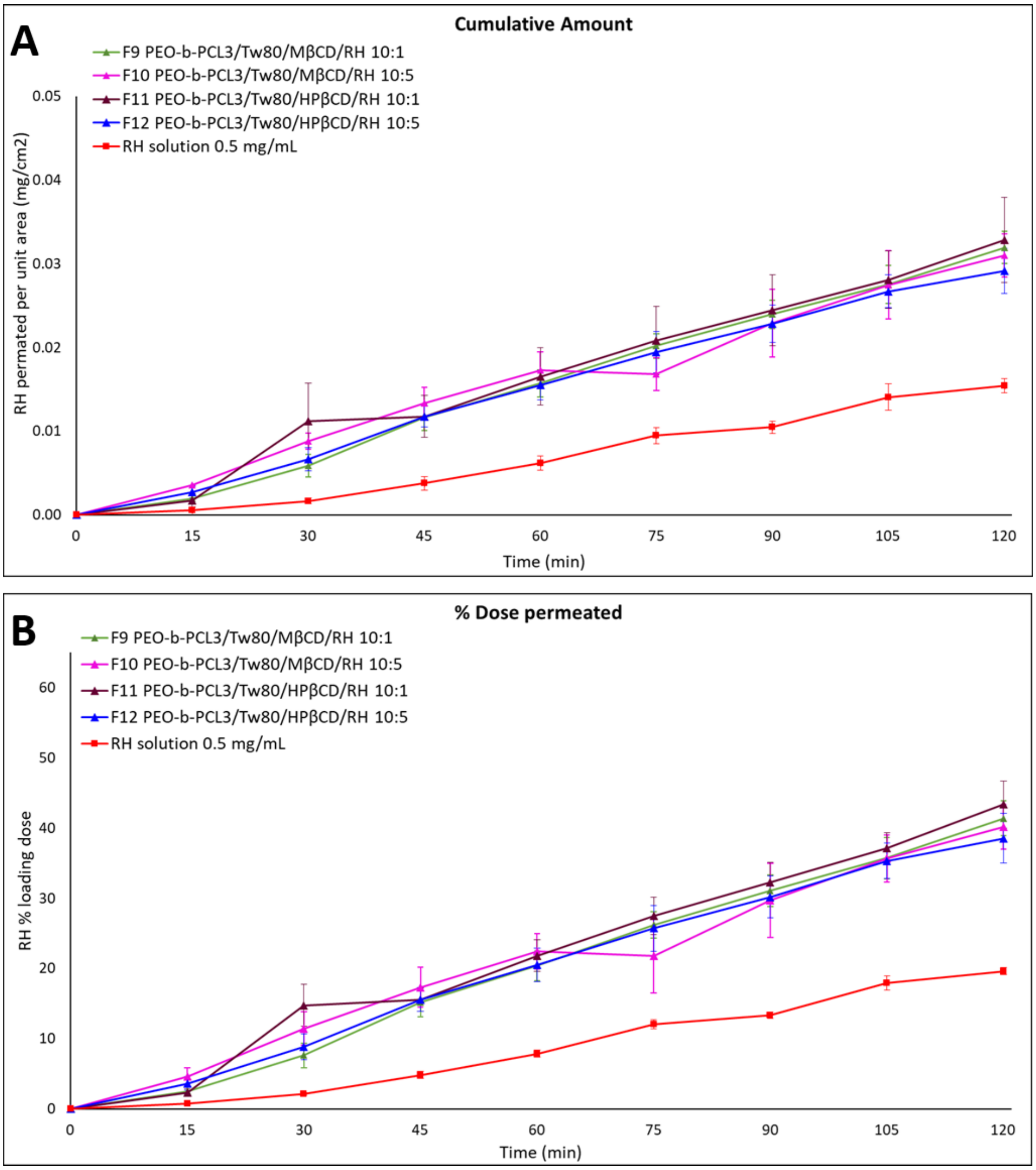

Figure S3. Permeation profiles of the colloidal dispersion of RH-loaded PEO-b-PCL_3 hybrid systems (F9–F12) at the dose of 0.05 mg through rabbit nasal tissue compared to a reference RH solution (0.5 mg/mL), expressed as (A) quantity permeated per unit area (mean ± SE, n = 4) and (B) % loading dose permeated for the tested formulation (mean ± SE, n = 4).

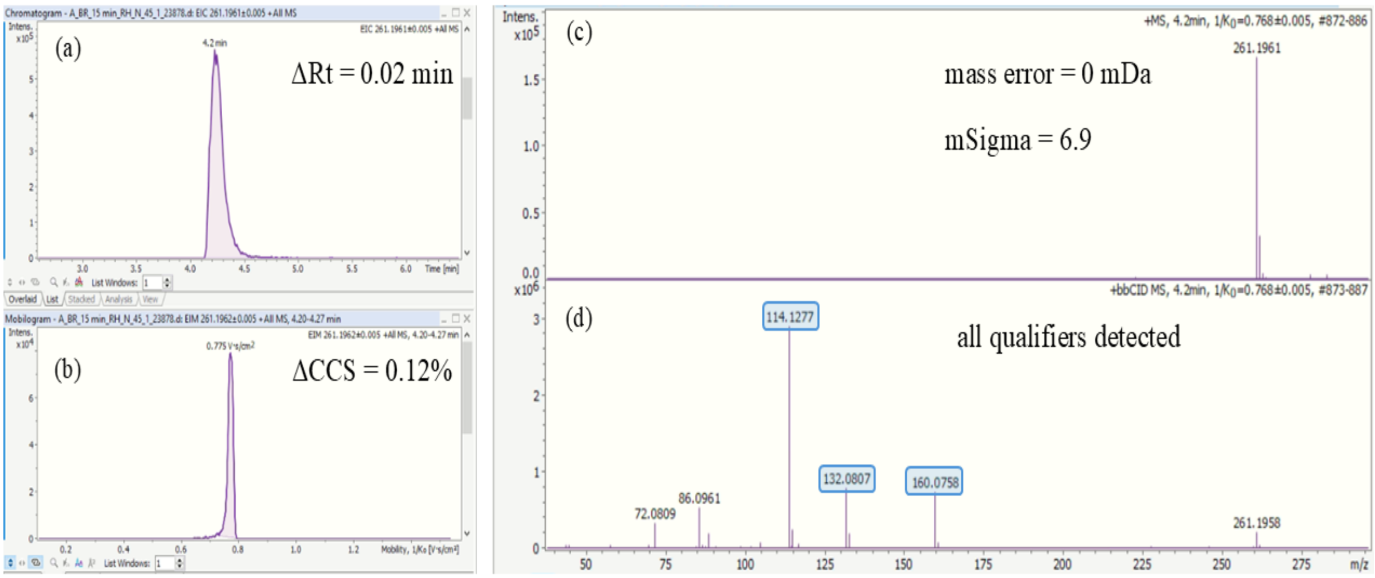

Figure S4. Ropinirole hydrochloride identification. a. EIC of RH with ΔR_t = 0.02 min , b. EIM of RH with ΔCCS = 0.12%, c. MS spectrum filtered based on the R_t and $1/K_0$ of RH (mass error = 0 mDa and isotopic fidelity of 6.9 mSigma), d. MS/MS spectrum filtered based on the Rt and $1/K_0$ of RH (all qualifiers were detected).
